# Psychoeducation Intervention Effectiveness to Improve Social Skills in Young People with ADHD: A Meta-Analysis

**DOI:** 10.1177/1087054721997553

**Published:** 2021-03-05

**Authors:** Lauren Amy Powell, Jack Parker, Anna Weighall, Valerie Harpin

**Affiliations:** 1 University of Sheffield, Sheffield, UK; 2 University of Derby, Derby, UK; 3 Ryegate Children’s Centre, Sheffield Children’s NHS Foundation Trust, Sheffield, UK

**Keywords:** attention deficit disorder with hyperactivity, education, social skills, children

## Abstract

**Objective:**

Attention Deficit Hyperactivity Disorder (ADHD) can be associated with limited understanding of the condition and poor social skills. Some evidence favors a psychoeducational approach, but little is known about the effectiveness of psychoeducation.

**Methods:**

Systematic review and meta-analysis of studies assessing psychoeducational interventions that aim to improve social skills of young people with ADHD.

**Results:**

Ten studies, including 943 participants, reported across 13 papers met the inclusion criteria. Although effect sizes were small, findings suggest the included interventions significantly improved social skills in young people with ADHD.

**Conclusions:**

Results show promise for psychoeducational behavioral interventions . However, the recommendations that can be developed from existing evidence are somewhat limited by the low quality of studies. Further rigorous trials are needed. In addition, future research should consider the long-term outcomes for these interventions, they should be iteratively co-designed and research should consider the context they intend to be delivered in.

## Introduction

Attention Deficit Hyperactivity Disorder (ADHD) is a common neurodevelopmental disorder affecting children and young people (CAYP) with a worldwide prevalence of around 3.4% of school age children (Polanczyk et al., 2015), 9.4% of the US population (Danielson et al., 2018) and 4.4% of adults (Brattberg, 2006). The Diagnostic Statistical Manual 5 (DSM-5) reports three core symptoms of ADHD; developmentally inappropriate levels of inattention, impulsivity and hyperactivity and distinguishes three presentations; predominantly inattentive, hyperactive/impulsive or combined (American Psychiatric Association (APA), 2013).

A number of difficulties have been reported in ADHD, such as executive dysfunction (Castellanos & Tannock, 2002), emotional dysfunction with low levels of emotional control (Anastopoulos et al., 2011), academic under achievement (DuPaul et al., 2011), increased likelihood of being expelled from school and leaving school early (McGee et al., 1991), poor social relationships and poor social functioning (Wehmeier et al., 2010).

Social functioning refers to social skills, information processing and peer functioning (Ros & Graziano, 2018). It is widely reported that CAYP with ADHD often display impaired social functioning (Willis et al., 2019). For example, CAYP with ADHD often show disruptive and developmentally inappropriate social behaviors and demonstrate less turn taking and cooperative behaviors compared to CAYP without ADHD (Barkley, 2014). CAYP with ADHD often display deficits in social communication skills (Bignell & Cain, 2007), social processing (Humphreys et al., 2016) and social cognition (Willis et al., 2019). Although CAYP attempt to have friendships with peers, these attempts are often viewed as negative, immature and intrusive (Ronk et al., 2011). CAYP with ADHD are also likely to be unaware of their impaired social skills (Owens, 2007) leading to difficulties maintaining peer relationships (Hoza et al., 2005).

ADHD is a highly co-morbid condition, particularly with Autism Spectrum Disorder (ASD) (National Institute for Health and Clinical Excellence (NICE), 2018). Indeed, CAYP with ASD present with similar social skills difficulties to CAYP with ADHD. Prior to the National Institute for Health and Care Excellence (NICE) guidelines (National Institute for Health and Clinical Excellence (NICE), 2008) and Diagnostic Statistical Manual 5 (DSM5) (American Psychiatric Association (APA), 2013), ASD was not recognized as a comorbid condition with ADHD. Clinical experience, however, demonstrated that there was, in fact, a significant overlap and more recent research has confirmed this (Ghirardi et al., 2018). Studies show that between 30 and 50% of individuals with ASD also show ADHD symptoms (particularly at pre-school age), and similarly, estimates suggest two-thirds of individuals with ADHD show features of ASD (Davis & Kollins, 2012). In a large Swedish cohort study (Ghirardi et al., 2018), it was found that 48% of those with ASD also fulfilled diagnostic criteria for ADHD and that 17% of those with ADHD had a diagnosis of ASD. It is important to note that findings from the Autism Treatment Network database suggest that co-occurrence of ADHD and ASD is associated with a lower quality of life and poorer adaptive functioning than in either of these conditions alone (Sikora et al., 2012).

Hence improving social skills in CAYP with ADHD and co-morbidities would be beneficial and psychoeducation is considered a possible intervention to achieve this.

Definitions of psychoeducation are heterogeneous. Initially, psychoeducation described a behavioral concept including briefing a patient about their illness, problem solving, communication and self-assertiveness training, which included relatives (Anderson et al., 1980). More recently, psychoeducation has been defined as interventions to teach individuals about their disorder by supporting them, providing information and disorder management skills (Bai et al., 2015) or, more simply, as “systematic and didactic approach to informing patients, and their relatives, about their illness and its treatment, thereby promoting understanding and personal management of the illness” (Ferrin et al., 2014).

Recipients of psychoeducation interventions can vary and include the individual, parents, teachers or others (Bai et al., 2015). Objectives of psychoeducation have been identified as learning about the disorder, facilitating informed disorder management and including the relative with this, patient empowerment and improving treatment adherence (Bäuml et al., 2006).

A plethora of evidence demonstrates the benefits of psychoeducation on adult populations (Willis et al., 2019). However, evidence also shows that psychoeducation could benefit young people with mood disorders (Cummings & Fristad, 2007; Fristad, 2006; Ginsburg et al., 2005). Regarding ADHD, it is argued that providing condition education, including a diagnostic label, can improve knowledge and attitudes in children and adults and that brief teacher training can improve knowledge and correct misconceptions of ADHD (Nussey et al., 2013). Evidence also favors providing age appropriate psychoeducation to CAYP with ADHD as a precursor to other formal treatment (Young et al., 2020) and suggests that parent education can improve treatment adherence in CAYP with ADHD (Nussey et al., 2013). Psychoeducation may enable the young person to become a partner in their ADHD treatment and improve their adherence to treatment (Wolraich et al., 2005).

Psychoeducation is recommended in a number of clinical guidelines for CAYP with ADHD for example, in the UK, NICE recommends psychoeducation for parents of CAYP with ADHD and for information to be provided to people with ADHD at a developmentally appropriate level, tailored to their individual needs (National Institute for Health and Clinical Excellence (NICE), 2018). The Canadian Clinical guidelines state that psychoeducation should empower patients and their families by providing information on the “. . .impact on daily functioning, treatment options, strategies for optimizing functioning” (Canadian ADHD Practice Guidelines, 2011). Similarly, the Spanish clinical guidelines recommend educational programs for parents, teachers and CAYP with ADHD (Guías de Práctica Clínica en el SNS, 2017).

Psychoeducation interventions that aim to benefit CAYP with ADHD can vary based on the form they take and the recipient of the intervention. For example, behavioral parent interventions are often based on social learning principles and include providing parents with strategies to reduce behavioral problems in their child and to improve parental attitudes toward parenting (Rimestad et al., 2019). The efficacy of parent interventions is also supported by meta-analysis data (Fabiano et al., 2009; Lee et al., 2012; Rimestad et al., 2019). However, concerns have been raised about the efficacy of parenting interventions in managing ADHD due to evidence that effect sizes drop to almost zero (Lee et al., 2012) when only data from blinded participants is analyzed (Sonuga-Barke et al., 2013). Larger effect sizes have however been reported in relation to parenting competence, which also moderately decrease over time and it has therefore been argued that exploration of sustainability of the effects of parental training over time requires further scrutiny (Lee et al., 2012). It should be noted that it can be challenging to blind participants when taking part in an RCT assessing a behavioral intervention.

Classroom-based interventions can include behavioral strategies for teachers and for example, promote the use of rewards to reduce problematic classroom behavior (Tarver et al., 2014) and focus on academic performance improvement (DuPaul et al., 2011). There is evidence in favor of classroom-based interventions (Tarver et al., 2014) and it appears that integration between home and school to ensure consistency with the behavioral approach is important (Raggi & Chronis, 2006).

Randomized controlled trial (RCT) evidence has demonstrated that psychoeducation interventions with families of CAYP with ADHD could help to reduce ADHD symptoms (Ferrin et al., 2016). Psychoeducation can also help people understand their condition and the treatment they receive leading to ownership of their treatment (Willis et al., 2019).

However, lack of adherence to treatments can weaken the impact of both pharmacological and psychosocial interventions (Bai et al., 2015), thus improving adherence is considered critical (Acri et al., 2018).

Interventions where CAYP with ADHD themselves are the recipients include child psychological therapy involving components such as social skills training, anger management and problem solving (Tarver et al., 2014). Evidence supporting these interventions is limited (Storebø et al., 2019) and little is known about the effectiveness of the psychoeducation mechanisms of the interventions and the impact they may specifically have upon the social skills of CAYP with ADHD.

Some review evidence does suggest that behavioral interventions (Fabiano et al., 2009) and social skills training can improve outcomes and social skills in CAYP with ADHD, respectively (Fabiano et al., 2009). Meta-analytic evidence shows improvements in social functioning resulting from peer involvement interventions (Cordier et al., 2018) however there was no specific assessment of psychoeducation included in these reviews.

A recent systematic review assessed psychoeducation interventions for parents and teachers of CAYP with ADHD (Dahl et al., 2020). This review concluded that psychoeducation can lead to improvements in ADHD symptoms and parent reported behavioral problems but did not assess the impact upon social skills in CAYP with ADHD or include interventions when the young person with ADHD is the recipient.

Therefore, this review aims to address the specific research question “Do psychoeducation interventions improve social skills in CAYP with ADHD?”

## Methods

The systematic review protocol was registered with PROSPERO (CRD42019157454) and was undertaken in accordance with the principles recommended in the Preferred Reporting Items for Systematic Reviews and Meta-Analysis (PRISMA) and the Meta-Analysis Reporting Standards (MARS) (Moher et al., 2009; APA Publications and Communications Board Working Group on Journal Article Reporting Standards, 2008).

### Search Methods

In line with the Cochrane Handbook (McKenzie et al., 2019) the Population Intervention Comparison Outcome Study Design (PICOS) framework helped to dictate the inclusion criteria and search terms for this review. The population for this review is CAYP with ADHD aged 18 years or under, the intervention is any intervention that aims to improve social skills (outcome) in CAYP with ADHD and included studies were RCTs only. The search terms were selected based upon the PICOS framework, Cochrane literature and information specialist advice.

Databases were searched from 1994 to 2019. This is because the Fourth edition of the Diagnostic Statistical Manual (DSM IV) (American Psychiatric Association, 1994) introduced the three subtypes of ADHD. Please note the current DSM edition DSM-5 (American Psychiatric Association (APA), 2013) was published in 2013. Authors decided publications since 1994 would be appropriate as changes made since DSM IV are subtle and including only publications since 2013 would significantly limit results presented in this review.

The search was conducted in the following databases in November 2019: MEDLINE, PsychINFO, The Cochrane Library, CINAHL, Web of Science (Core Collection), ProQuest, ASSIA and Scopus. Medical Subject Headings (MeSH) keywords used were child, child behavior, adolescent, young adult, adolescent health, adolescent psychiatry, students, minors, young adult, attention deficit and disruptive behavior disorders, attention deficit disorder with hyperactivity, conduct disorder, attention, hyperkinesis, patient education as topic, education, health education, teaching, schools, training support, knowledge, patient medication knowledge, behavior, adolescent behavior, behavior control, behavior therapy, child behavior, problem behavior, behavioral research, behavioral symptoms, attitude, attitude to health, social skills, social behavior, interpersonal relations, social isolation, social problems, social skills, peer group, communication, interpersonal relations, friends.

Text terms used were child disorders, young people, young person, teenage, student, school age, minor, boy, girl, YP, teen, youth, young, juvenile, Juvenescent, pubescent, conduct disorders, child behavior disorders, ADHD, ADDH, ADHS, HKD, TDAH, behave, disrupt, disorder, defiant, impulsive, inattentive, inattention, psychoeducation, educate, education medical, train, teach, school, tuition, tutor, coach, guide, instruct, inform, knowledge, develop, lesson, behavior change, behavioral, conduct, disruptive, impulse control and conduct disorders, habit, prosocial, interact, social, social develop, disrupt, peer reject, communicate, empathy, peer problem, peer interact, Social dysfunction, Peer relationship, peer function, peer reject, friendship.

Terms were combined using Boolean logic (“AND,” “OR”). MeSH are specific recognized terms used to identify journal articles and books in electronic databases. Free text terms and synonyms are specific words that the search strategy looks for in the title and abstract.

The MEDLINE search strategy is available in Supplemental Appendix 1. Electronic references were downloaded to reference management software.

For the purpose of this review, psychoeducation is defined as an intervention which “*includes information about the illness and its treatment, skills development, and patient empowerment*” (Montoya et al., 2011).

This approach is consistent with a recent review (Dahl et al., 2020). As a result, studies included in this review deliver psychoeducation in a variety of formats, to a variety of audiences with differing content and modes of delivery.

The inclusion criteria are outlined in Table 1. Details of the included outcome measures can be found in Supplemental Appendix 2.

**Table 1.** Inclusion/Exclusion Criteria for this Review.

Inclusion criteria	Exclusion criteria
• Intervention must contain a psychoeducational component based on the definition provided above • Intervention must focus on social skill development in CAYP with ADHD • Intervention must aim to benefit CAYP with ADHD 18 years or under • Intervention can be undertaken by anybody (e.g., parents, child, teachers) as long as the beneficiary is a young person with ADHD • Must have a “pure” control group diagnosed with ADHD that is, control group does not receive any other reported intervention within the study other than usual care • Primary research testing an intervention • Study must be an RCT • Paper written in English language • Must measure social skills of CAYP with ADHD aged 18 or under • Studies published after 1994 (see “Search Methods” above for rationale) • Participants reported to have obtained a clinical diagnosis of ADHD	• Not aimed to benefit CAYP with ADHD • Not an interventional study • Secondary research for example, a review • Control group receives more than usual care • Not research • Study does not include an arm whereby psychoeducation is being received • Intervention does not have a psychoeducational component • Intervention does not aim to improve social skills in CAYP with ADHD • Paper not in English language • Studies published before 1994 • Studies not measuring social skills in CAYP with ADHD • Participants not reported to have obtained a clinical diagnosis of ADHD

### Quality Assessment

The methodological quality of the included RCTs was assessed using the Cochrane Risk of Bias Tool (CRoB) (Higgins & Altman, 2008). This tool addresses fields including sequence generation, allocation concealment, blinding of participants and personnel, blinding of outcome assessment, incomplete outcome data, and selective outcome reporting. RCTs were stated as having either a low risk of bias if they were rated as low for three key areas: allocation concealment, blinding of outcome assessment and completeness of outcome data. They were stated to have an overall high risk of bias of any of these three key areas were judged as having a high risk of bias. RCTs stated to have an overall unclear risk of bias were so if any of the three key areas were stated to be unclear. Quality of evidence of the trials was also assessed according to the Grading of Recommendations Assessment, Development and Evaluation (GRADE) approach using GRADE Pro software. GRADE provides a robust and transparent framework for presenting summaries of evidence, providing a systematic approach to making clinical practice recommendations. It is a widely used tool for evaluating the reliability of the evidence with over 100 organizations worldwide officially endorsing GRADE. The use of this framework ensures rigorous and replicable assessment of the quality of evidence and enable decisions to be made about the relative weight that should be given to included studies when developing recommendations for practice (Brignardello-Petersen et al., 2018). AW assessed the quality of evidence and LP and JP checked the assessment.

### Data Extraction

Titles, abstracts, and/or full text papers were screened independently by two review authors (LP, JP) to identify studies compliant with the inclusion criteria. Reviewers resolved disagreements through discussion. A standardized Microsoft Excel form was used to extract data. Details of the study characteristics, including location of study, participants, the intervention, comparator and results were recorded. Data extraction was carried out by reviewer LP and checked for accuracy by reviewers AW and JP.

### Data Synthesis

A random effects meta-analysis and narrative review was undertaken with tables and text providing supporting evidence. Revman5 (The Cochrane Collaboration, 2014) was used to conduct the meta-analysis. A random effect size model with 95% confidence intervals was adopted and effect sizes calculated to indicate intervention efficacy to improve social skills in CAYP with ADHD. Data required for the meta-analysis was extracted by author LP and checked for accuracy by authors JP and AW.

## Results

### Summary

**Figure 1.**
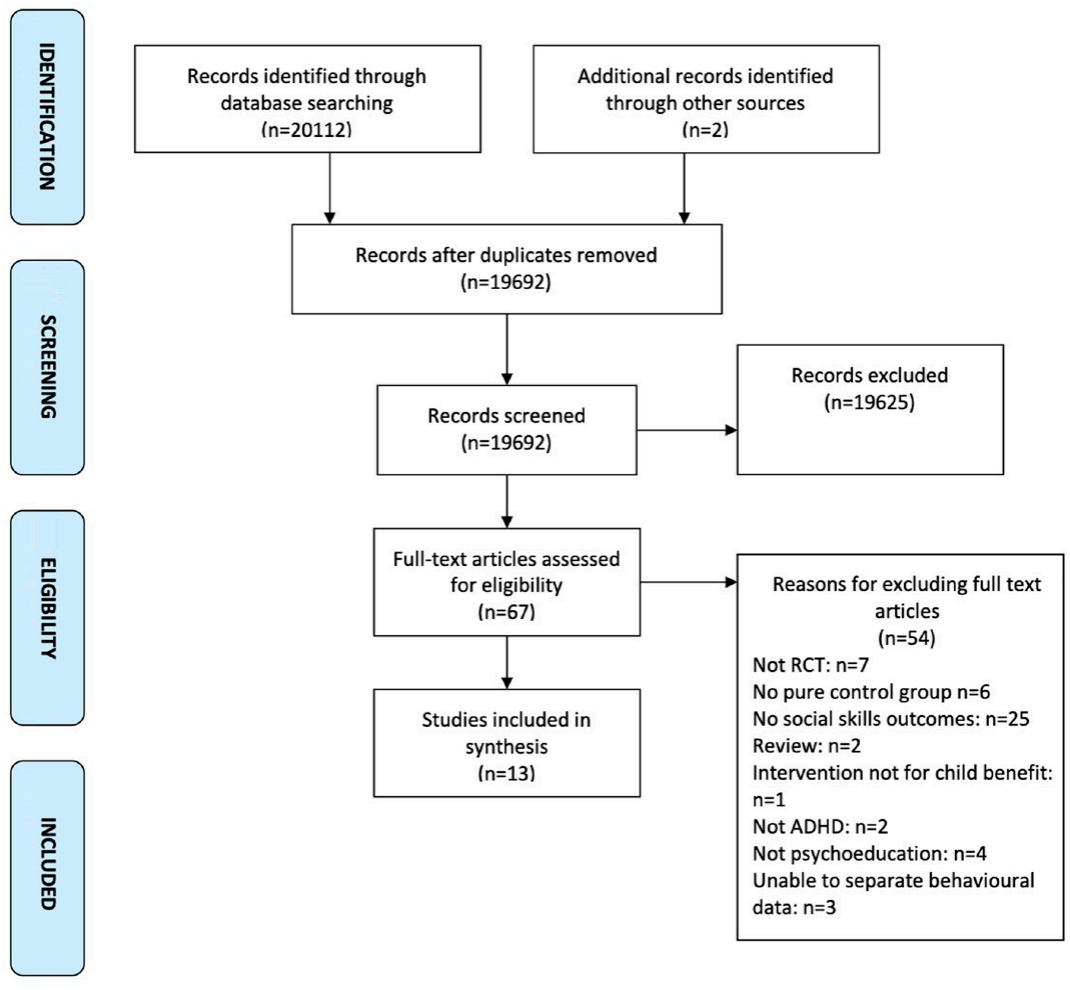
Search results.

The electronic literature search yielded a total of 20112 records following deduplication. Two additional citations were identified via handpicking methods. This involved reviewing reference sections of papers during the selection process. Therefore 20114 citations were screened by authors LP and JP and 19625 articles were excluded based upon information in the titles and abstracts. At this stage, 67 full texts were obtained, 54 were excluded (see Figure 1) and 13 obtained to be included in this review. Ten studies were reported across the 13 included articles.

Overall, there were 943 CAYP with ADHD recruited across all included studies (*n* = 10) with 886 participants at follow up. This means there was a mean of 94 participants at baseline and 89 at follow up with a mean of just five participants dropping out per study.

The 10 included studies included one from the UK (Ferrin et al., 2016), one from Sweden (Östberg & Rydell, 2012), one from Australia (Wilkes-Gillan et al., 2016) and the remaining 10 were conducted in North America (Chacko et al., 2009; Haack et al., 2017; Mikami et al., 2010; Pfiffner & McBurnett, 1997; Pfiffner et al., 2007, 2014, 2016, 2018; Webster-Stratton et al., 2011, 2013). Collectively, all included studies involved 943 participants at baseline and 886 at follow up including control groups, equating to an average of a 6.04% dropout overall. Child participants had a mean age of 8.6 (range: 5.3–10.95).

### Medication Status

One study stipulated ADHD medication must be “stable” for at least 1 month before they took part in the study (Ferrin et al., 2016), one did not report whether CAYP with ADHD were medicated (Pfiffner et al., 2007), one trial stipulated that their participants must not be taking ADHD medication (Webster-Stratton et al., 2011, 2013) and the remaining trials simply reported the percentage of CAYP with ADHD who were medicated when they were recruited (Chacko et al., 2009; Haack et al., 2017; Mikami et al., 2010; Östberg & Rydell, 2012; Pfiffner & McBurnett, 1997; Pfiffner et al., 2014, 2016, 2018; Wilkes-Gillan et al., 2016).

Eight of the 10 trials observed significant improvements following the intervention in social skills in CAYP with ADHD (Haack et al., 2017; Mikami et al., 2010; Östberg & Rydell, 2012; Pfiffner & McBurnett, 1997; Pfiffner et al., 2007, 2014, 2016, 2018; Webster-Stratton et al., 2011, 2013; Wilkes-Gillan et al., 2016).

### Comorbidities

Three of the included studies did not report on comorbidities of their participants (Chacko et al., 2009; Pfiffner & McBurnett, 1997; Pfiffner et al., 2016, 2018), one excluded CAYP with ADHD that had diagnoses of any “major developmental disorder” (Wilkes-Gillan et al., 2016). One study reported on comorbid oppositional defiant disorder (ODD) only (Webster-Stratton et al., 2011, 2013), and two studies reported on comorbid anxiety, depression and ODD (Haack et al., 2017; Mikami et al., 2010; Pfiffner et al., 2014). The remaining three studies fully reported on comorbid conditions (Ferrin et al., 2016; Östberg & Rydell, 2012; Pfiffner et al., 2007).

### Outcomes

**Figure 2.**
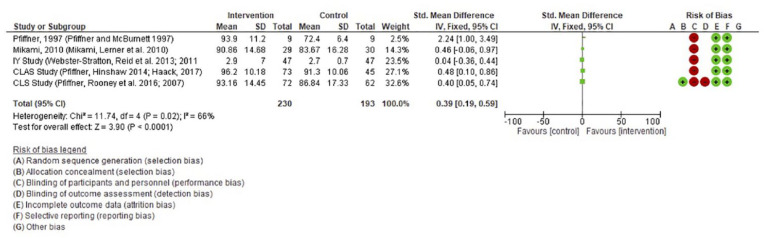
Meta-analysis of parent reported outcomes of social skills in CAYP with ADHD. Included in this meta-analysis were five studies reported across eight papers.

**Figure 3.**
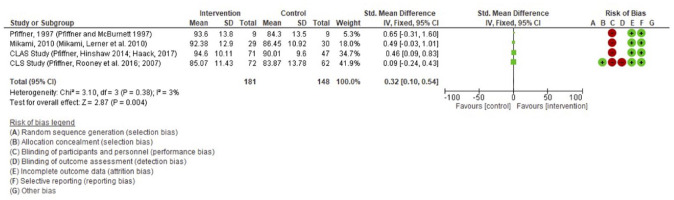
Meta-analysis of teacher reported outcomes of social skills in CAYP with ADHD. Four studies reported across six papers were included in this meta-analysis.

The results of the meta-analysis are presented graphically in Figures 2 and 3. Three parent and teacher reported outcome measures (Social skills rating system: SSRS; Social skills improvement system: SSIS; Social competence scale: SCS) were included in the analysis. This means that for the meta-analyses of parent reported and teacher reported outcome measures, five studies reported across eight papers and four studies reported across six papers were included, respectively. The Incredible years study (Webster-Stratton et al., 2011, 2013) was included in the parent but not the teacher reported outcome meta analyses as they did not adopt a suitable teacher outcome measure. The remaining five studies were not included in the meta-analysis as they also did not report on suitable outcome measures to be fairly compared with the other studies.

### Risk of Bias and Meta-Analyses

The left column of the figures provides the author and date of the relevant study. The means, standard deviations (SD) and weight of each study is then provided in the following columns for the intervention and control groups. The vertical line through the forest plot gives the 95% confidence interval. On the right-hand side of each figure, there is a summary of the CRoB results.

The meta -analysis found significant between group differences in favor of the intervention for improving social skills in CAYP with ADHD for both the teacher reported (*p* = .004) and parent reported measures (*p* = .0001). The effect size is also significant but small for both parent (.39) and teacher (.32) measures.

### Interventions Aimed at a Single Audience (CAYP with ADHD or Parents)

Only one study reported an intervention whereby CAYP with ADHD are the only recipients of the intervention (Wilkes-Gillan et al., 2016). Significant between group differences were observed compared to a control group for the Test of Playfulness outcome measure.

Three interventions across three articles reported RCTs whereby parents are the only recipient of the intervention (Chacko et al., 2009; Ferrin et al., 2016; Mikami et al., 2010). Two of the three studies found no significant differences between groups for social skills. (Chacko et al., 2009; Ferrin et al., 2016). One of the three studies found that Parental Friendship Coaching predicted improved parent reported child social skills post-test (*p* < .01) using the SSRS however, these results were not supported by teacher rated SSRS scores (Mikami et al., 2010).

According to the parent reported Quality of Play questionnaire, Parental Friendship Coaching was associated with reductions in the amount of conflict (*p* < .01) and the amount of disengagement displayed by children on playdates (*p* = .52) (Mikami et al., 2010). This measure involved teaching parents to structure their child’s playdates to optimize their child and friend’s social interaction and was therefore felt appropriate to include (Mikami et al., 2010).

### Interventions Aimed at Multiple Audiences

Two studies reported interventions across three articles that targeted both children and parents in separate groups (Pfiffner & McBurnett, 1997; Webster-Stratton et al., 2011, 2013), one study reported an intervention across two articles that included parents and children in separate groups but also included a classroom component (Pfiffner et al., 2016, 2018), one study reported an intervention involving parents groups, groups that involved the parent, child, therapist and teachers as well as a child group (Pfiffner et al., 2007), one study reported an intervention across two articles involved family meetings and teacher consultations (Haack et al., 2017; Pfiffner et al., 2014). The final study involved groups for parents and also meetings with teachers (Östberg & Rydell, 2012). Of the six studies reported above, all reported improvements in social skills.

One study observed improved parent ratings of the SDQ (*p* < 0.05) with problematic behaviors reducing only in the intervention group. Prosocial behavior improvements in the SDQ were not observed (Östberg & Rydell, 2012).

Pfiffner and McBurnett (1997) study showed improved SSRS and UCI parent rated social skills in those who undertook Social Skills Training (SST) and Parent mediated SST compared to the control group and these effects were maintained at a 4-month follow-up (*p* < .0001). However, teacher rated SSRS scores did not demonstrate a significant improvement in social skills (*p* > .1). The parent mediated SST group also demonstrated improved social skills as reported by the teacher rated SSRS from pre-treatment to post-treatment (*p* .001) and from pre-treatment to follow up (*p* < .001). Significant differences were not found between the SST and control groups (*p* > .1) (Pfiffner & McBurnett, 1997).

A further study by Pfiffner et al. (2007) found significantly improved parent and teacher ratings on the SSRS between groups post-treatment, (*p* = .0065). The Test of Life Skills Knowledge also found significant between group differences, favoring the intervention group, for knowledge of social and organizational skills taught during the intervention (*p* = .0001) (Pfiffner et al., 2007).

The CLS study (Pfiffner et al., 2016, 2018) found significant between group differences post-treatment favoring the intervention as reported in the social skills subscale of the SSIS (*p* = .0393). They also found significant between group differences in favor of the CLAS intervention for both parent-reported (*p* = 0.04) and teacher reported (*p* = 0.02) SSIS. Differences were maintained at follow up but not then significant for teacher reported outcomes.

The Incredible Years Study (Webster-Stratton et al., 2011, 2013) found significant improvements in the Wally Problem Solving Test within the intervention arm 1-year post treatment (*p* < .001).

### Quality Appraisal

**Figure 4.**
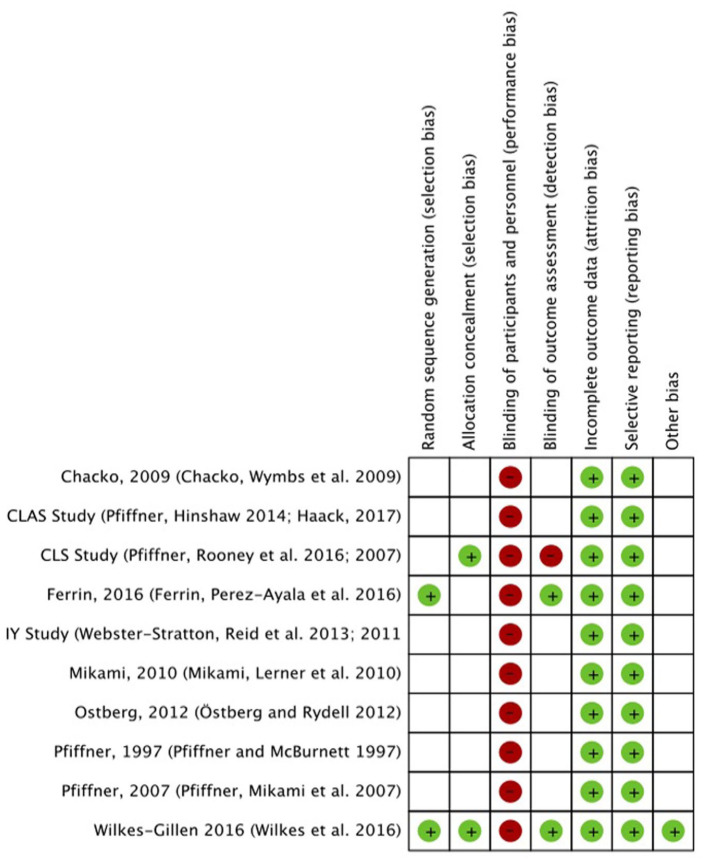
Risk of bias summary: review authors’ judgments about each risk of bias item for each included study.

**Figure 5.**
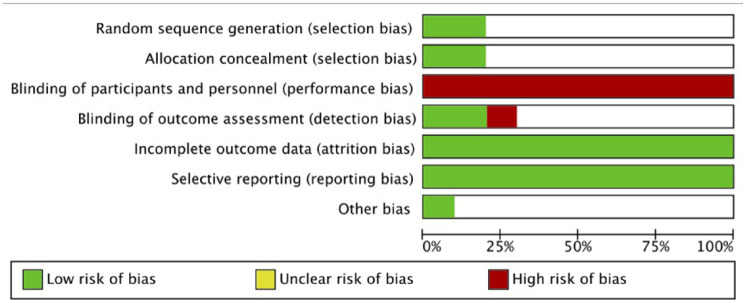
Risk of bias graph: review authors’ judgments about each risk of bias item presented as percentages across all included studies.

The CRoB quality assessment summary can be found in Figures 4 and 5 and further details of the full CRoB quality appraisal can be found in Supplemental Appendix 3. One of the 10 included studies was judged has having an overall low risk of bias (Wilkes-Gillan et al., 2016). One of the studies was judged as having an overall high risk of bias as a result of having a high risk of bias for the blinding of outcome assessment domain (Pfiffner et al., 2016, 2018). The remaining eight studies were judged as having an overall unclear risk of bias (Chacko et al., 2009; Ferrin et al., 2016; Haack et al., 2017; Mikami et al., 2010; Östberg & Rydell, 2012; Pfiffner & McBurnett, 1997; Pfiffner et al., 2007, 2014; Webster-Stratton et al., 2011, 2013). It should be noted that all studies gained a high risk of bias in terms of blinding of participants. This is a challenge in studies of this type of intervention.

Parent and teacher outcomes were assessed using the GRADE quality assessment and found that there was on average, a low quality of evidence. This means that any conclusions and recommendations should be viewed with caution and further high-quality research is needed. Table 2 shows the results of this analysis and Table 3 summarizes the included studies in this review.

**Table 2.** Summary of Findings and Quality Assessment Table.

Certainty assessment							No. of patients		Effect		Certainty	Importance
No.studies	Study design	Risk of bias	Inconsistency	Indirectness	Imprecision	Other considerations	Interventions to improve social skills	comparator	Relative (95% CI)	Absolute (95% CI)		
Social skills: parent												
5	Randomized trials	Serious^a,b^	Not serious	Not serious	Serious^c^	None	230	193	–	SMD 0.39 SD higher (0.19 higher to 0.59 higher)	⨁⨁◯◯ Low	Critical
Social skills: teachers												
4	Randomized trials	Serious^a,d^	Not serious	Not serious	Serious^c^	None	181	148	–	SMD 0.32 higher (0.1 higher to 0.54 higher)	⨁⨁◯◯ Low	Important

*Note.* CI = confidence interval; SMD = standardized mean difference.

a Unclear risk of bias across most included trials.

b Parents in all included trials knew which group their children belonged to and this could have led to a detection bias.

c Small overall sample size.

d Teachers in all included trials knew which group children belonged to and this could have led to a detection bias.

**Table 3.** Study Summary Table.

Author, date, country, study design and arm descriptions	Number participants *N* (*n*); number of follow up time points and timeframes	Gender, mean age (child); Participants medicated	Intervention name, length, frequency, who undertakes intervention	Control description	Outcome measures assessed; who completed measures, results and *p* values
Chacko et al. (2009), America; 3 arm RCT	*N* (*n*) = 120 (115)	STEPP: 77% male; BPT: 66% male, control: 69% Male; STEPP: 7.36; BPT: 8.17; control: 8.02; STEPP: 40%; BPT: 35%; control: 37.5%; determined by study staff, based upon DSM criteria. 20% of STEPP children and 10% of	Strategies to enhance positive parenting (STEPP); STEPP: 2 hours a week over 9 weeks, parents and children	Traditional behavior parent training (BPT) and waitlist group. BPT: 2 hours weekly, 9 weeks	Impairment rating scale—parents; no between or within group statistically significant results observed for the IRS
Ferrin et al. (2016), United Kingdom; 2 arm RCT	*N* (*n*) = 69 (62). Intervention: 35 (32 6 weeks, 31 6 months); control 34 (30 6 weeks, 28 6 months); baseline 6 week and 6-month follow ups	Psychoeducation group: 29M, 6F; control: 31M, 3F; psychoeducation group: 10.86; control: 10.56	Psychoeducation; 6 × 2-hour sessions once a week, parents; 12 parenting therapist led sessions	Treatment as usual: continued routine medical care	Strengths and difficulties questionnaire (SDQ)—parent, teacher and child versions; no between or within group statistically significant results observed for the parent, teacher or child rated SDQ
CLAS Study, (Haack et al., 2017; Pfiffner et al., 2014); America; 3 arm RCT	*N* (*n*) = 199 (187). CLAS (intervention): 74 (73 post treatment, 69 follow up); PFT: 74 (74 post treatment, 73 follow up); Control: 51 (48 post treatment, 45 follow up); baseline, post treatment and 5–7 months follow up	CLAS: 38M, 36F, PFT 48M, 26F; TAU: 30M, 21F; 8.6: CLAS: 8.8; PFT: 8.7; TAU: 8.4; CLAS: 9.5%; PFT: 1.4%; TAU: 2%	Child life and attention skills treatment (CLAS); 10 × 90-minute parent groups, 6 × 30-minute family meetings, 10 × 90-minute child group meetings, teacher consultation including 30-minute meeting, 5 × 30-minute teacher meetings, parent, child and therapist	Treatment as usual	Social skills improvement system rating scales (SSIS)—teachers and parents; significant between group differences observed in favor of the intervention (CLAS) for both parent reported (*p* = 0.04) and teacher reported (*p* = 0.02) SSIS
Mikami et al. (2010), America; Pilot 2 arm RCT	*N* (*n*) = 124 (119). PFC 32 (29 post-test, 28 follow up), ADHD control 30 (30 post-test, 29 follow up), normative comparison 62 (62); baseline, post-test, 1-month follow up	PFC: 21M, 11F; ADHD control: 21M, 9F, normative comparison: 42M, 20F; PFC: 8.28, ADHD control: 8.23; normative comparison: 8.23; PFC and ADHD control: 24.8%	Parental friendship coaching (PFC); 8 × 90-minute sessions, once weekly	No treatment control group, also included a normative comparison group	SSRS—parents and teachers; quality of play questionnaire (QPQ)—parents SSRS: PFC predicted higher parent reports of child’s social skills post-test (*p* = 0.04). No significant results reported for teacher rated SSRS. QPQ: receipt of PFC associated with reductions in amount of conflict (*p* < 0.01) and amount of disengagement (*p* < 0.01) children displayed on playdates; reported by parents
Östberg et al. (2012), Sweden; 2 arm RCT	Parents: *N* (*n*) = 70(65): intervention: 36 (30 post-test, 29 follow up); control 34 (24 post-test, 32 follow up). Teachers: *N* (*n*) = 77 (70): intervention: 38 (38 post-test, 35 follow up); control: 39 (38 post-test, 35 follow up); baseline, post-test, 3-month follow up	Intervention: 25M, 4F; control: 26M, 6F; intervention: 11.1; control: 10.8; intervention: 86%; control: 77%	Strategies in everyday life; parents 10 weekly 2 hours sessions, teachers: 8 sessions	Pure control group, participants in this group received intervention at the end of the study	SDQ—parent and teachers; there was one significant effect on parent ratings of the SDQ-total (*p* < 0.05) with problematic behaviors reducing only in the intervention group. No significant differences were reported with regards to the prosocial behavior items of the SDQ
Pfiffner and McBurnett (1997), America; 3 arm RCT	*N* (*n*) = 27(27): SST-PG: 9 (9 post-test and follow up); SST: 9 (9 post-test and follow up); waitlist: 9 (9 post-test and follow up), baseline, posttreatment, 4-month follow up	19M, 8F: SST-PG: 6M, 3F; SST: 6M, 3F; waitlist: 7M, 2F; aged 8–10 years	Child SST and SST-PG; 8 weekly 90-minutes sessions	Wait list control group, received SST-PG after the follow up measures were taken	SSRS—parents and teachers; UCI—parents; test of social skill knowledge—child; both groups combined significantly improved parent reports of social skills compared to control group. Effects maintained at 4-month follow up (SSRS: *p* < 0.0001). Improvements compared to control group observed in pooled treatment groups for UCI (*p* < 0.0001). Teacher rated SSRS not significant (*p* > 0.1) SST-PG treatment group showed significant teacher rated SSRS scores from pre-treatment to posttreatment (*p* 0.015) and from pre-treatment to follow up (*p* < 0.01). Scores for SST and control groups did not significantly change from pre-treatment to post-treatment (*p* > 0.1)
Pfiffner et al. (2007), America; 2 arm RCT	*N* (*n*) = 69 (54). Intervention: 36 (36 post-test, 29 follow up); control 33 (30 post-test, 25 follow up); baseline, post-test and variable timescales for follow ups	46M, 23F; CLAS: 8.8; PFT: 8.7; control: 8.4	CLAS: 12 weeks. Teachers: initial 30 minutes followed by 4–5 30 minutes meetings with teachers, parent child, therapist	Control group (“business as usual”) did not receive the intervention, offered intervention at end of study	SSRS—parents and teachers; test of life skill knowledge—child; SSRS: parent and teacher ratings of social functioning showed significant between group differences at post-treatment favoring the treatment group (*p* = 0.0065). Test of life skills knowledge: children’s knowledge of social and organizational skills taught during the group showed significant between group differences favoring the intervention group (*p* = 0.0001)
Collaborative life skills training (CLS) study (Pfiffner et al., 2016, 2018) America; 2 arm cluster RCT intervention: collaborative life skills (CLS) and control (“Business as usual; BAU”).	*N* (*n*) = 135 (134). Intervention: 72 (72 post-test and follow up); control: 63 (62 post-test and follow up); baseline, posttreatment, follow up next school year	CLS: 54M, 18F; control 42M, 21F; CLS: 8.3; control: 8.5; CLS: 9.7%; control: 7.9%	CLS classroom: teachers attended 1 × 1-hour session, 1 × 30-minute meeting and 2–3 individual 30 minutes meetings with teacher, parent and child	Usual services (in schools), intervention not received	Social skills improvement system—parents and teachers; parent ratings of SSIS showed significant between group differences posttreatment favoring the CLS group (*p* = 0.0034). Teachers ratings of the SSIS were not statistically significant
Incredible years (IY) study (Webster Stratton et al., 2011, 2013) America; 2 arm RCT	*N* (*n*) = 99 (94). IY: 49 (47 post-test, 42 follow up); Control:50 (47 post-test; follow up not applicable for controls). Baseline; post-test, 1 year follow up	IY: 36M, 13F; control: 39M, 11F; IY: 5.3; control: 5.3; no medication at randomization	Incredible years (IY; parents) and IY dinosaur program (children); 20 weekly 2 hours group sessions for both IY and IY dinosaur programs	Waitlist control condition	Social competence scale—parents; wally problem solving test (WPST)—children; significant between group differences were reported for the SCS (*p* < 0.001) and the WPST (*p* < 0.01) pre and post intervention. Significant improvements in the wally problem solving test were observed within the intervention arm 1-year post-treatment (*p* < 0.001) and no significant differences were observed within the treatment group for the social competence scale
Wilkes-Gillen et al. (2016), Australia; 2 group parallel RCT	*N* (*n*) = 31 (29). Intervention: 16 (15 post-test, 15 at post-test and follow up); control 15 (14 at post-test and follow up); baseline, post-test, 1-month follow up	Intervention: 13M, 2F; control: 12M; 2F; intervention: 8.2; control: 8.5; intervention: 9 medicated; control: 11 medicated	Play based intervention; 6 clinic play sessions for child and weekly home modules delivered by parent	Waitlist condition, received intervention after 10 weeks of wait time	Test of playfulness—children (observation); significant between group differences were observed in favor of the intervention group for the test of playfulness (*p* < 0.001)

### Meta-Analysis Results

Two meta-analyses were conducted. Of the 10 included studies, eight were included in the meta-analysis.

## Discussion

### Summary of Results

This review set out to answer the question “Do psychoeducation interventions improve social skills in CAYP with ADHD?” Following exclusions, 10 studies reported across 13 articles were included. Overall, there were 943 CAYP with ADHD recruited across all 10 included studies with 886 participants at follow up. This means there was a mean of 94 participants at baseline and 89 at follow up with a mean average of five participants dropping out per study.

Encouragingly, our meta-analysis indicated small but significant improvements in social skills in CAYP with ADHD in favor of the intervention for both parent and teacher reported outcome measures. Seven of the included 10 studies involved CAYP with ADHD as recipients of the intervention (Chacko et al., 2009; Haack et al., 2017; Pfiffner & McBurnett, 1997; Pfiffner et al., 2007, 2014, 2016, 2018; Webster-Stratton et al., 2011, 2013; Wilkes-Gillan et al., 2016). Six of these eight studies reported significant improvements in social skills in CAYP with ADHD (Chacko et al., 2009; Haack et al., 2017; Pfiffner & McBurnett, 1997; Pfiffner et al., 2007, 2014, 2016, 2018; Webster-Stratton et al., 2011, 2013; Wilkes-Gillan et al., 2016). These six studies all engaged CAYP interactively in the intervention through activities such as group work, role-play, problem solving, coaching, behavioral rehearsal and feedback. This could indicate that CAYP with ADHD are more likely to have a preference toward an interactive learning style.

One of the eight studies that included children as intervention recipients did not report significant improvements in social skills in CAYP with ADHD (Chacko et al., 2009). This study concentrated on behavioral impairment rather than ADHD symptoms. This is important because although their participants included CAYP with ADHD, it was not the difficulties with social skills that resulted directly from the ADHD that were being measured.

### Contribution to Knowledge

Recent systematic review evidence (Dahl et al., 2020) has concluded that psychoeducation interventions for parents and teachers can lead to improvements in behavior in CAYP with ADHD and that there is little evidence in favor of behavioral interventions improving peer social functioning in CAYP with ADHD (Morris et al., 2020). This review adds to the existing evidence base by only including studies evaluating interventions that involve a psychoeducational component and specifically assessing the impact these interventions have upon social skills in CAYP with ADHD.

### Outcome Measures

Our meta-analysis shows that parent reported measures were likely to demonstrate a significant improvement in social skills in CAYP with ADHD. It should be acknowledged, however, that parents were not blinded to the intervention their children were receiving. This could indicate that parents, who would have invested their time and effort into the interventions, expected or hoped to observe improvements in their child’s social skills and could therefore reflect observer bias. It should, however, be noted that due to the nature of the interventions, it is often impossible to blind participants and their families to the arm of the study allocated to the participant. The meta-analysis also demonstrated that the teacher reported measures were significant in improving social skills for CAYP with ADHD, adding weight to the parent reported measure findings.

It is clear that social skills difficulties are a significant problem for CAYP with ADHD and their families. However, previous research as to the value of psychoeducation in establishing long term improvements in social functioning in CAYP with ADHD remains difficult for clinicians to interpret. The studies reported in this review involved significant time commitment from parents, teachers and clinicians.

Although 10 studies are included in this review, they include a large number of, often non-comparable, outcome measures (*n* = 15). This is why five of the 10 included studies were not included in the meta-analysis. It is important to note that the interventions included in this review do not focus solely on psychoeducation therefore results should be interpreted with caution as it is difficult to definitively state that the effectiveness of the interventions in the meta-analysis are due to the psychoeducation mechanisms provided.

Further, it would be beneficial if consistent outcome measures could be adopted across multiple trials to enable fair comparison across studies. In order to reduce bias, it may be useful to additionally adopt social skills assessments that do not involve subjective measures in order to decrease the potential for bias by the evaluators. This could involve the utilization of emerging objective measures such as the use of technology (Hult et al., 2018; Muñoz-Organero et al., 2019).

### Exploring Psychoeducation and Social Skills Training

This review does however highlight the potential for interventions for CAYP with ADHD to include a psychoeducational component that educates the child about ADHD and/or teaches them a new skill to help them cope with their ADHD related difficulties such as social skills training. This approach has been successful in other conditions such as depression in adolescents (Jones et al., 2018), asthma in CAYP (Marsland et al., 2019) and epilepsy in adolescents (Snead et al., 2004), as well as in young people with ADHD (Dahl et al., 2020). The clinical significance of these findings favoring psychoducation for CAYP with ADHD are particularly important as evidence suggests that those treated with psychoeducation as well as another treatment such as medication, tend to have better treatment acceptance, adherence and better long-term outcomes (Adler & Nierenberg, 2010; Bai et al., 2015). Further, interventions that educate the parent were included in this review. Evidence suggests that parent knowledge of ADHD can lead to improved treatment outcomes for their child due to the parents’ improved confidence in enrolling their child in behavioral and psychological treatments (MacKay & Corkum, 2006).

Further research is required to explore which components should comprise such interventions for CAYP with ADHD in order to make them successful.

It must however be noted that research to evaluate interventions to improve social skills in CAYP with ADHD is complex. ADHD is a highly variable, comorbid condition and the individuals involved often live in complex circumstances. No single intervention will work for every child.

A recent systematic review of 25 studies reported across 45 papers concluded that “there is little evidence to support or refute social skills training for children and adolescents with ADHD” (Storebø et al., 2019). It must be noted that the Storebø review focused on social skills training and not psychoeducational mechanisms, as in the case of this present review. However other research suggests that there is in fact limited benefits of social skills training (Mikami et al., 2014). Mikami et al. (2014) argued that traditional clinic-based social skills training may be ineffective because it focuses on teaching social skills knowledge without addressing the performance barriers that prevent the young people from using the knowledge gained in practical ways. It was hypothesized that social skills training is ineffective because it fails to consider factors that contribute to impaired relationships between children with ADHD and their peers such as different peer attitudes, exclusionary behaviors and negative attitudes toward young people with ADHD. However, not all social skills interventions both generally and in this review are traditional clinic-based interventions. This is important as applying knowledge in practice in varying contexts is key.

In the future, design of social skills interventions should consider both the variable personal and environmental context in which the intervention delivered (World Health Organisation (WHO), 2018). That is, studies investigating these interventions should aim to answer the question “what works for whom under what circumstances and respects?” (Pawson et al., 1997). This would enable resources to be targeted optimally. This can be achieved via methodologies such as Realist Evaluation (RE) that aims to explore the mechanisms underpinning an intervention (Bonell et al., 2012). Having an in depth understanding of the theoretical mechanisms underpinning the intervention and what components work, for whom and in what circumstances could improve the outcome of the intervention (Rycroft-Malone et al., 2010).

### What Psychoeducation is Needed?

Social skills training interventions for CAYP with ADHD are often based upon the assumption that CAYP with ADHD do not understand social skills and need to be taught what they are. This often happens in a clinic setting that is not representative of real life scenarios, hence the argument that social skills training will not work in such an artificial situation (Mikami et al., 2014). However, it has been reported that there may not be a deficit in CAYP with ADHD acquiring social skills knowledge, but a deficit may exist whereby the young person is unable to perform social skills (Aduen et al., 2018). That is, understanding social sills may not be problematic, but putting them into practice may be. To this end, future psychoeducational social skills interventions for CAYP with ADHD may therefore wish to educate CAYP with ADHD around how to put their social skills into practice in real life situations to help enhance social skills performance, rather than only teaching them what social skills are.

It is also important to consider that there is no way of knowing the extent to which comorbid conditions have confounded the extent to which CAYP with ADHD may respond to the interventions in this review. This is especially challenging as three of the 10 included RCTs did not report on comorbid conditions and one RCT excluded participants who had a diagnosis of any other “major developmental disorder” (Wilkes-Gillan et al., 2016). This is important because it has also been reported that different presentations of ADHD and different comorbidities such as Oppositional Defiant Disorder (ODD) may present different social problem profiles (De Boo & Prins, 2007). Therefore, it is proposed that not only should future social skills psychoeducation for CAYP with ADHD to focus upon putting social skills into practice, but it should also be tailored to the individual and their needs. Future research should also report on all of the comorbidities of their participants.

### Limitations and Future Research Recommendations

Of the 10 included studies, only one was conducted in the UK; one in Sweden, one in Australia and seven studies in North America. Therefore, the conclusions of this review must be generalized to an international population with caution. The included studies are also limited to those provided in the English language, which can cause information bias.

The evidence is limited to the broad definition of psychoeducation adopted (Montoya et al., 2011). This was due to the heterogeneous definitions of psychoeducation in the literature. Future research may wish to address this and work toward a standardized definition of psychoeducation to guide clinicians. As with all RCTs of behavioral interventions, blinding can be problematic as can the inclusion of a pure control group due to naturally occurring confounds that cannot be controlled.

This review is also limited to the evidence in the literature, which as previously discussed, runs the risk of not being representative of a wider population of families who live with CAYP with ADHD and have difficulty accessing such support. Much of the evidence does not consider maintenance of efficacy and ongoing support needs. Future research is advised to consider this (DuPaul et al., 2020) and to explore innovative ways by which ongoing support could be provided to CAYP with ADHD and their families.

### Reporting

Future research should report significant factors that could impact upon the effectiveness of their intervention. This includes gender, the presentation and severity of participants’ ADHD, whether or not a parent has previously attended a parent group, socio-demographic factors, comorbid conditions including ASD and if the child is taking ADHD medication at any point in the study. Where possible, this information should at least be collected at baseline and at the end of the study to highlight any changes during the duration of the study. If a young person is optimized on medication, does this improve outcomes of psychoeducation and indeed other behavioral therapies?

### Patient Empowerment through Co-Design

Psychoeducational interventions for CAYP have the potential to empower the individual and to maximize self-care (Barlow & Ellard, 2004) and offer the possibility of the need for less medication. They may also lay the foundations for improved outcomes in adult life. However, in order to increase the likelihood that the intervention will lead to impact (i.e., achieve the desired outcome), it is important that they are designed properly from the outset (DuPaul et al., 2020). Co-design methodologies involving the end users and stakeholders at every stage of intervention development are recommended to achieve this (Blower et al., 2020; Greenhalgh et al., 2016).

Co-design can be a challenging approach, especially when working with a population with attention difficulties such as ADHD. However, evidence suggests that it is indeed possible (Fekete & Lucero, 2019; Powell et al., 2017). However, careful consideration for the mechanisms and components of design are essential to understand what component of subsequent treatment is leading to better social outcomes. Fekete and Lucero (2019) report a number of recommendations on how to effectively co-design with this population.

### Conclusions

ADHD is a complex, comorbid condition. Individuals with ADHD can benefit from a package of care which includes a number of interventions targeting different facets of their difficulties (National Institute for Health and Clinical Excellence (NICE), 2018).

The findings of this review indicate that behavioral interventions including a psychoeducation element could be valuable for improving social skills in CAYP with ADHD. The effect sizes in the present review are small but significant. Specifically, involving CAYP with ADHD interactively in the intervention shows promise and may be a reflection of CAYP with ADHD requiring support with the performance rather than the acquisition of social skills. However, the quality of the included studies is uniformly, limited so the conclusion should be generalized with caution. Blinding of the participant and their families is often impossible when delivering behavioral interventions within an RCT design. It would be beneficial if consistent outcome measures and optimal study design to reduce bias could be agreed.

We recommend that future interventions to improve social skills in CAYP with ADHD should involve a psychoeducational component, clear and transparent reporting, be co-designed, and consider the personal and environmental contexts in which the intervention is to be delivered. Only then can clinicians understand which interventions will best support the complex children, young people and families they strive to support.

## Supplemental Material

sj-pdf-1-jad-10.1177_1087054721997553 – Supplemental material for Psychoeducation Intervention Effectiveness to Improve Social Skills in Young People with ADHD: A Meta-Analysis Click here for additional data file. Supplemental material, sj-pdf-1-jad-10.1177_1087054721997553 for Psychoeducation Intervention Effectiveness to Improve Social Skills in Young People with ADHD: A Meta-Analysis by Lauren Amy Powell, Jack Parker, Anna Weighall and Valerie Harpin in Journal of Attention Disorders
