# Supplementary material for: Psychoeducation Intervention Effectiveness to Improve Social Skills in Young People with ADHD: A Meta-Analysis
Source: J Atten Disord. 2021 Mar 5;26(3):340–57. doi: 10.1177/1087054721997553 (PMC8785297; doi:10.1177/1087054721997553)
Supplement: sj-pdf-1-jad-10.1177_1087054721997553 – Supplemental material for Psychoeducation Intervention Effectiveness to Improve Social Skills in Young People with ADHD: A Meta-Analysis [file sj-pdf-1-jad-10.1177_1087054721997553.pdf]

Appendices:

Appendix 1: Medline Search Strategy

Database(s): **Ovid MEDLINE(R) and Epub Ahead of Print, In-Process & Other Non-Indexed Citations and Daily** 1946 to January 17, 2020

Search Strategy:

#	Searches	Results
1	exp Child Behavior/ or child*.mp.	2387314
2	child*.tw.	1343963
3	young people.mp.	26170
4	young people.tw.	25964
5	young person.mp.	1023
6	young person.tw.	1011
7	teenage*.mp.	20917
8	teenage*.tw.	20663
9	student.mp. or exp Students/	175619
10	student*.tw.	268585
11	school age*.mp.	21855
12	school age.tw.	12896
13	minor*.mp.	302938
14	minor*.tw.	287214

15	boy*.mp.	150521
16	boy*.tw.	150166
17	girl*.mp.	144862
18	girl*.tw.	144795
19	YP.mp.	1323
20	YP.tw.	1317
21	teen*.mp.	30005
22	teen*.tw.	29583
23	youth*.mp.	77732
24	youth*.tw.	70942
25	young*.mp.	1373034
26	young*.tw.	656895
27	Juvenile*.mp.	90617
28	juvenile*.tw.	79238
29	juvenescent*.mp.	8
30	juvenescent*.tw.	8
31	pubescent*.mp.	841
32	pubescent*.tw.	838
33	young adult*.mp.	866107
34	young adult*.tw.	91002

35	1 or 2 or 3 or 4 or 5 or 6 or 7 or 8 or 9 or 10 or 11 or 12 or 13 or 14 or 15 or 16 or 17 or 18 or 19 or 20 or 21 or 22 or 23 or 24 or 25 or 26 or 27 or 28 or 29 or 30 or 31 or 32 or 33 or 34	4021877
36	(attention deficit and disruptive behavio?r disorders).mp. [mp=title, abstract, original title, name of substance word, subject heading word, floating sub-heading word, keyword heading word, organism supplementary concept word, protocol supplementary concept word, rare disease supplementary concept word, unique identifier, synonyms]	3043
37	attention deficit disorder with hyperactivity.mp. or exp Attention Deficit Disorder with Hyperactivity/	28019
38	attention deficit disorder with hyperactivity.tw.	239
39	exp Attention Deficit Disorder with Hyperactivity/ or exp Conduct Disorder/ or conduct disorder*.mp.	34910
40	conduct disorder*.tw.	4534
41	ADHD.mp.	24346
42	ADHD.tw.	23852
43	ADDH.mp.	118
44	ADDH.tw.	116
45	ADHS.mp.	683
46	ADHS.tw.	543
47	HKD.mp.	145
48	HKD.tw.	144

49	TDAH.mp.	126
50	TDAH.tw.	70
51	exp Attention/ or attention*.mp.	447584
52	attention*.tw.	402224
53	behav*.mp.	1656875
54	behav*.tw.	1225598
55	disrupt*.mp.	284831
56	disrupt*.tw.	278072
57	disorder*.mp.	1941915
58	disorder*.tw.	1078583
59	defian*.mp.	2808
60	defian*.tw.	2748
61	impulsiv*.mp.	21811
62	impulsiv*.tw.	19640
63	inattentiv*.mp.	2337
64	inattentiv*.tw.	2333
65	inattention*.mp.	5289
66	inattention*.tw.	5209
67	hyperkinesis.mp. or exp Hyperkinesis/	4717
68	hyperkinesis.tw.	715
69	hyperkin*.mp.	8144

70	hyperkin*.tw.	4743
71	36 or 37 or 38 or 39 or 40 or 41 or 42 or 43 or 44 or 45 or 46 or 47 or 48 or 49 or 50 or 51 or 52 or 53 or 54 or 55 or 56 or 57 or 58 or 59 or 60 or 61 or 62 or 63 or 64 or 65 or 66 or 67 or 68 or 69 or 70	3840668
72	Psychoeducation.mp. or exp Psychoeducation/	2802
73	psychoeducation.tw.	2674
74	exp Medical Education/ or exp Early Intervention/ or educat*.mp.	1012979
75	educat*.tw.	563413
76	train*.mp.	546383
77	train*.tw.	512371
78	exp Teaching/ or teach*.mp.	250007
79	teach*.tw.	183544
80	school*.mp.	311203
81	school*.tw.	272586
82	tuition*.mp.	774
83	tuition.tw.	758
84	tutor*.mp.	10983
85	tutor*.tw.	10604
86	coach*.mp.	13882
87	coach*.tw.	13714
88	guide*.mp.	753986

89	guide*.tw.	633730
90	instruct*.mp.	103371
91	instruct*.tw.	91779
92	inform*.mp.	1504837
93	inform*.tw.	1398790
94	knowledg*.mp.	739452
95	knowledge*.tw.	672629
96	develop*.mp.	4747235
97	develop*.tw.	4186655
98	lesson*.mp.	60812
99	lesson*.tw.	60779
100	72 or 73 or 74 or 75 or 76 or 77 or 78 or 79 or 80 or 81 or 82 or 83 or 84 or 85 or 86 or 87 or 88 or 89 or 90 or 91 or 92 or 93 or 94 or 95 or 96 or 97 or 98 or 99	7789452
101	behavio?r*.mp.	1615367
102	behavio?r*.tw.	1182939
103	exp Behavior/ or exp Behavior Therapy/ or exp Adaptive Behavior/ or exp Prosocial Behavior/ or exp Child Behavior/	1870050
104	behavio?r change*.mp.	17139
105	behavio?r change*.tw.	15907
106	behavio?ral*.mp.	390398

107	behavio?ral*.tw.	350627
108	exp Behavior Problems/ or exp Conduct Disorder/ or conduct*.mp.	1322352
109	conduct*.tw.	1246323
110	exp Habits/ or habit*.mp.	183677
111	habit*.tw.	170123
112	attitude*.mp.	412515
113	attitude*.tw.	143049
114	101 or 102 or 103 or 104 or 105 or 106 or 107 or 108 or 109 or 110 or 111 or 112 or 113	4076909
115	exp Social Skills/ or social skill*.mp.	6238
116	social skill*.tw.	5069
117	prosocial*.mp.	4521
118	prosocial*.tw.	4353
119	interact*.mp.	1628091
120	interact*.tw.	1489500
121	exp Social Skills/ or exp Social Skills Training/ or exp Social Isolation/ or social*.mp.	764153
122	social develop*.mp.	4337
123	social develop*.tw.	3221
124	disrupt*.mp.	284831
125	disrupt*.tw.	278072

126	peer reject*.mp.	402
127	peer reject*.tw.	395
128	communicat*.mp.	402742
129	communicat*.tw.	280581
130	exp Empathy/ or empath*.mp.	27355
131	empath*.tw.	14855
132	peer problem*.mp.	559
133	peer problem*.tw.	557
134	peer interact*.mp.	764
135	peer interact*.tw.	745
136	social dysfunction*.mp.	1157
137	social dysfunction*.tw.	1155
138	peer relationship*.mp.	1658
139	peer relationship*.tw.	1570
140	peer function*.mp.	55
141	peer function*.tw.	55
142	peer reject*.mp.	402
143	peer reject*.tw.	395
144	exp Friendship/ or friendship*.mp.	8641
145	friendship*.tw.	5054

146	115 or 116 or 117 or 118 or 119 or 120 or 121 or 122 or 123 or 124 or 125 or 126 or 127 or 128 or 129 or 130 or 131 or 132 or 133 or 134 or 135 or 136 or 137 or 138 or 139 or 140 or 141 or 142 or 143 or 144 or 145	2874962
147	35 and 71 and 100 and 114 and 146	123228
148	limit 147 to (human and english language and (childhood or adolescence <13 to 17 years>) and (160 preschool age or 180 school age) and human and yr="1994 - 2019") [Limit not valid in Ovid MEDLINE(R),Ovid MEDLINE(R) Daily Update,Ovid MEDLINE(R) In-Process,Ovid MEDLINE(R) Publisher; records were retained]	88196
149	148 not autism.mp. [mp=title, abstract, original title, name of substance word, subject heading word, floating sub-heading word, keyword heading word, organism supplementary concept word, protocol supplementary concept word, rare disease supplementary concept word, unique identifier, synonyms]	82247
150	149 not ASD.mp. [mp=title, abstract, original title, name of substance word, subject heading word, floating sub-heading word, keyword heading word, organism supplementary concept word, protocol supplementary concept word, rare disease supplementary concept word, unique identifier, synonyms]	82172
151	150 not cerebral palsy.mp. [mp=title, abstract, original title, name of substance word, subject heading word, floating sub-heading word, keyword heading word, organism supplementary concept word, protocol supplementary concept word, rare disease supplementary concept word, unique identifier, synonyms]	81806

152	151 not tourettes.mp. [mp=title, abstract, original title, name of substance word, subject heading word, floating sub-heading word, keyword heading word, organism supplementary concept word, protocol supplementary concept word, rare disease supplementary concept word, unique identifier, synonyms]	81751
153	152 not sex.mp. [mp=title, abstract, original title, name of substance word, subject heading word, floating sub-heading word, keyword heading word, organism supplementary concept word, protocol supplementary concept word, rare disease supplementary concept word, unique identifier, synonyms]	69276
154	153 not anxiety.mp. [mp=title, abstract, original title, name of substance word, subject heading word, floating sub-heading word, keyword heading word, organism supplementary concept word, protocol supplementary concept word, rare disease supplementary concept word, unique identifier, synonyms]	63978
155	154 not depression.mp. [mp=title, abstract, original title, name of substance word, subject heading word, floating sub-heading word, keyword heading word, organism supplementary concept word, protocol supplementary concept word, rare disease supplementary concept word, unique identifier, synonyms]	60140
156	155 not depress*.mp. [mp=title, abstract, original title, name of substance word, subject heading word, floating sub-heading word, keyword heading word, organism supplementary concept word, protocol supplementary concept word, rare disease supplementary concept word, unique identifier, synonyms]	59340
157	156 not sex*.mp. [mp=title, abstract, original title, name of substance word, subject heading word, floating sub-heading word, keyword heading word,	55652

	organism supplementary concept word, protocol supplementary concept word, rare disease supplementary concept word, unique identifier, synonyms]	
158	157 not diabetes.mp. [mp=title, abstract, original title, name of substance word, subject heading word, floating sub-heading word, keyword heading word, organism supplementary concept word, protocol supplementary concept word, rare disease supplementary concept word, unique identifier, synonyms]	54696
159	158 not fetal*.mp. [mp=title, abstract, original title, name of substance word, subject heading word, floating sub-heading word, keyword heading word, organism supplementary concept word, protocol supplementary concept word, rare disease supplementary concept word, unique identifier, synonyms]	54131
160	159 not HIV.mp. [mp=title, abstract, original title, name of substance word, subject heading word, floating sub-heading word, keyword heading word, organism supplementary concept word, protocol supplementary concept word, rare disease supplementary concept word, unique identifier, synonyms]	53364
161	160 not cross section*.mp. [mp=title, abstract, original title, name of substance word, subject heading word, floating sub-heading word, keyword heading word, organism supplementary concept word, protocol supplementary concept word, rare disease supplementary concept word, unique identifier, synonyms]	48346
162	161 not soldier*.mp. [mp=title, abstract, original title, name of substance word, subject heading word, floating sub-heading word, keyword heading word, organism supplementary concept word, protocol supplementary concept word, rare disease supplementary concept word, unique identifier, synonyms]	48302

163	162 not case stud*.mp. [mp=title, abstract, original title, name of substance word, subject heading word, floating sub-heading word, keyword heading word, organism supplementary concept word, protocol supplementary concept word, rare disease supplementary concept word, unique identifier, synonyms]	47594
164	163 not suicide.mp. [mp=title, abstract, original title, name of substance word, subject heading word, floating sub-heading word, keyword heading word, organism supplementary concept word, protocol supplementary concept word, rare disease supplementary concept word, unique identifier, synonyms]	47098
165	164 not malaria.mp. [mp=title, abstract, original title, name of substance word, subject heading word, floating sub-heading word, keyword heading word, organism supplementary concept word, protocol supplementary concept word, rare disease supplementary concept word, unique identifier, synonyms]	46951
166	165 not obes*.mp. [mp=title, abstract, original title, name of substance word, subject heading word, floating sub-heading word, keyword heading word, organism supplementary concept word, protocol supplementary concept word, rare disease supplementary concept word, unique identifier, synonyms]	45051
167	166 not protocol.mp. [mp=title, abstract, original title, name of substance word, subject heading word, floating sub-heading word, keyword heading word, organism supplementary concept word, protocol supplementary concept word, rare disease supplementary concept word, unique identifier, synonyms]	44406
168	167 not respiratory*.mp. [mp=title, abstract, original title, name of substance word, subject heading word, floating sub-heading word, keyword heading	44023

	word, organism supplementary concept word, protocol supplementary concept word, rare disease supplementary concept word, unique identifier, synonyms]	
169	168 not methylphenidate.mp. [mp=title, abstract, original title, name of substance word, subject heading word, floating sub-heading word, keyword heading word, organism supplementary concept word, protocol supplementary concept word, rare disease supplementary concept word, unique identifier, synonyms]	43857
170	169 not medicat*.mp. [mp=title, abstract, original title, name of substance word, subject heading word, floating sub-heading word, keyword heading word, organism supplementary concept word, protocol supplementary concept word, rare disease supplementary concept word, unique identifier, synonyms]	42834
171	170 not cancer.mp. [mp=title, abstract, original title, name of substance word, subject heading word, floating sub-heading word, keyword heading word, organism supplementary concept word, protocol supplementary concept word, rare disease supplementary concept word, unique identifier, synonyms]	41680
172	171 not autis*.mp. [mp=title, abstract, original title, name of substance word, subject heading word, floating sub-heading word, keyword heading word, organism supplementary concept word, protocol supplementary concept word, rare disease supplementary concept word, unique identifier, synonyms]	41414
173	172 not reproduc*.mp. [mp=title, abstract, original title, name of substance word, subject heading word, floating sub-heading word, keyword heading	39429

	word, organism supplementary concept word, protocol supplementary concept word, rare disease supplementary concept word, unique identifier, synonyms]	
174	173 not vitamin.mp. [mp=title, abstract, original title, name of substance word, subject heading word, floating sub-heading word, keyword heading word, organism supplementary concept word, protocol supplementary concept word, rare disease supplementary concept word, unique identifier, synonyms]	39338
175	174 not endocrine.mp. [mp=title, abstract, original title, name of substance word, subject heading word, floating sub-heading word, keyword heading word, organism supplementary concept word, protocol supplementary concept word, rare disease supplementary concept word, unique identifier, synonyms]	39246
176	175 not violen*.mp. [mp=title, abstract, original title, name of substance word, subject heading word, floating sub-heading word, keyword heading word, organism supplementary concept word, protocol supplementary concept word, rare disease supplementary concept word, unique identifier, synonyms]	37799
177	176 not quasi.mp. [mp=title, abstract, original title, name of substance word, subject heading word, floating sub-heading word, keyword heading word, organism supplementary concept word, protocol supplementary concept word, rare disease supplementary concept word, unique identifier, synonyms]	37476
178	177 not music*.mp. [mp=title, abstract, original title, name of substance word, subject heading word, floating sub-heading word, keyword heading word, organism supplementary concept word, protocol supplementary concept word, rare disease supplementary concept word, unique identifier, synonyms]	37220

179	178 not review.mp. [mp=title, abstract, original title, name of substance word, subject heading word, floating sub-heading word, keyword heading word, organism supplementary concept word, protocol supplementary concept word, rare disease supplementary concept word, unique identifier, synonyms]	32600
180	179 not cohort.mp. [mp=title, abstract, original title, name of substance word, subject heading word, floating sub-heading word, keyword heading word, organism supplementary concept word, protocol supplementary concept word, rare disease supplementary concept word, unique identifier, synonyms]	30941
181	180 not overweight.mp. [mp=title, abstract, original title, name of substance word, subject heading word, floating sub-heading word, keyword heading word, organism supplementary concept word, protocol supplementary concept word, rare disease supplementary concept word, unique identifier, synonyms]	30827
182	181 not psycho*.mp. [mp=title, abstract, original title, name of substance word, subject heading word, floating sub-heading word, keyword heading word, organism supplementary concept word, protocol supplementary concept word, rare disease supplementary concept word, unique identifier, synonyms]	11024
183	182 not PTSD.mp. [mp=title, abstract, original title, name of substance word, subject heading word, floating sub-heading word, keyword heading word, organism supplementary concept word, protocol supplementary concept word, rare disease supplementary concept word, unique identifier, synonyms]	11020
184	183 not alcohol.mp. [mp=title, abstract, original title, name of substance word, subject heading word, floating sub-heading word, keyword heading word,	10501

	organism supplementary concept word, protocol supplementary concept word, rare disease supplementary concept word, unique identifier, synonyms]	
185	184 not feasibility.mp. [mp=title, abstract, original title, name of substance word, subject heading word, floating sub-heading word, keyword heading word, organism supplementary concept word, protocol supplementary concept word, rare disease supplementary concept word, unique identifier, synonyms]	10338
186	185 not AIDS.mp. [mp=title, abstract, original title, name of substance word, subject heading word, floating sub-heading word, keyword heading word, organism supplementary concept word, protocol supplementary concept word, rare disease supplementary concept word, unique identifier, synonyms]	10206
187	186 not postnatal.mp. [mp=title, abstract, original title, name of substance word, subject heading word, floating sub-heading word, keyword heading word, organism supplementary concept word, protocol supplementary concept word, rare disease supplementary concept word, unique identifier, synonyms]	10156
188	187 not infect*.mp. [mp=title, abstract, original title, name of substance word, subject heading word, floating sub-heading word, keyword heading word, organism supplementary concept word, protocol supplementary concept word, rare disease supplementary concept word, unique identifier, synonyms]	9951
189	188 not immun*.mp. [mp=title, abstract, original title, name of substance word, subject heading word, floating sub-heading word, keyword heading word, organism supplementary concept word, protocol supplementary concept word, rare disease supplementary concept word, unique identifier, synonyms]	9819

190	189 not surg*.mp. [mp=title, abstract, original title, name of substance word, subject heading word, floating sub-heading word, keyword heading word, organism supplementary concept word, protocol supplementary concept word, rare disease supplementary concept word, unique identifier, synonyms]	9586
191	190 not dent*.mp. [mp=title, abstract, original title, name of substance word, subject heading word, floating sub-heading word, keyword heading word, organism supplementary concept word, protocol supplementary concept word, rare disease supplementary concept word, unique identifier, synonyms]	9273
192	191 not smok*.mp. [mp=title, abstract, original title, name of substance word, subject heading word, floating sub-heading word, keyword heading word, organism supplementary concept word, protocol supplementary concept word, rare disease supplementary concept word, unique identifier, synonyms]	8865
193	192 not pilot.mp. [mp=title, abstract, original title, name of substance word, subject heading word, floating sub-heading word, keyword heading word, organism supplementary concept word, protocol supplementary concept word, rare disease supplementary concept word, unique identifier, synonyms]	8595
194	193 not pregnan*.mp. [mp=title, abstract, original title, name of substance word, subject heading word, floating sub-heading word, keyword heading word, organism supplementary concept word, protocol supplementary concept word, rare disease supplementary concept word, unique identifier, synonyms]	8314
195	194 not postpartum.mp. [mp=title, abstract, original title, name of substance word, subject heading word, floating sub-heading word, keyword heading	8308

	word, organism supplementary concept word, protocol supplementary concept word, rare disease supplementary concept word, unique identifier, synonyms]	
196	195 not brief report.mp. [mp=title, abstract, original title, name of substance word, subject heading word, floating sub-heading word, keyword heading word, organism supplementary concept word, protocol supplementary concept word, rare disease supplementary concept word, unique identifier, synonyms]	8297
197	196 not oral*.mp. [mp=title, abstract, original title, name of substance word, subject heading word, floating sub-heading word, keyword heading word, organism supplementary concept word, protocol supplementary concept word, rare disease supplementary concept word, unique identifier, synonyms]	8192
198	197 not prison.mp. [mp=title, abstract, original title, name of substance word, subject heading word, floating sub-heading word, keyword heading word, organism supplementary concept word, protocol supplementary concept word, rare disease supplementary concept word, unique identifier, synonyms]	8186
199	198 not newborn*.mp. [mp=title, abstract, original title, name of substance word, subject heading word, floating sub-heading word, keyword heading word, organism supplementary concept word, protocol supplementary concept word, rare disease supplementary concept word, unique identifier, synonyms]	7931
200	199 not pain*.mp. [mp=title, abstract, original title, name of substance word, subject heading word, floating sub-heading word, keyword heading word, organism supplementary concept word, protocol supplementary concept word, rare disease supplementary concept word, unique identifier, synonyms]	7834

201	200 not tumor*.mp. [mp=title, abstract, original title, name of substance word, subject heading word, floating sub-heading word, keyword heading word, organism supplementary concept word, protocol supplementary concept word, rare disease supplementary concept word, unique identifier, synonyms]	7822
202	201 not anorexia*.mp. [mp=title, abstract, original title, name of substance word, subject heading word, floating sub-heading word, keyword heading word, organism supplementary concept word, protocol supplementary concept word, rare disease supplementary concept word, unique identifier, synonyms]	7813
203	202 not bulimia.mp. [mp=title, abstract, original title, name of substance word, subject heading word, floating sub-heading word, keyword heading word, organism supplementary concept word, protocol supplementary concept word, rare disease supplementary concept word, unique identifier, synonyms]	7812
204	203 not eat* disorder*.mp. [mp=title, abstract, original title, name of substance word, subject heading word, floating sub-heading word, keyword heading word, organism supplementary concept word, protocol supplementary concept word, rare disease supplementary concept word, unique identifier, synonyms]	7786
205	204 not asperger*.mp. [mp=title, abstract, original title, name of substance word, subject heading word, floating sub-heading word, keyword heading word, organism supplementary concept word, protocol supplementary concept word, rare disease supplementary concept word, unique identifier, synonyms]	7783
206	205 not suicid*.mp. [mp=title, abstract, original title, name of substance word, subject heading word, floating sub-heading word, keyword heading word,	7776

	organism supplementary concept word, protocol supplementary concept word, rare disease supplementary concept word, unique identifier, synonyms]	
207	206 not bipolar.mp. [mp=title, abstract, original title, name of substance word, subject heading word, floating sub-heading word, keyword heading word, organism supplementary concept word, protocol supplementary concept word, rare disease supplementary concept word, unique identifier, synonyms]	7757
208	207 not schizo*.mp. [mp=title, abstract, original title, name of substance word, subject heading word, floating sub-heading word, keyword heading word, organism supplementary concept word, protocol supplementary concept word, rare disease supplementary concept word, unique identifier, synonyms]	7700
209	208 not infant*.mp. [mp=title, abstract, original title, name of substance word, subject heading word, floating sub-heading word, keyword heading word, organism supplementary concept word, protocol supplementary concept word, rare disease supplementary concept word, unique identifier, synonyms]	6961
210	209 not dyslexi*.mp. [mp=title, abstract, original title, name of substance word, subject heading word, floating sub-heading word, keyword heading word, organism supplementary concept word, protocol supplementary concept word, rare disease supplementary concept word, unique identifier, synonyms]	6915
211	210 not traum*.mp. [mp=title, abstract, original title, name of substance word, subject heading word, floating sub-heading word, keyword heading word, organism supplementary concept word, protocol supplementary concept word, rare disease supplementary concept word, unique identifier, synonyms]	6826

212	211 not phobi*.mp. [mp=title, abstract, original title, name of substance word, subject heading word, floating sub-heading word, keyword heading word, organism supplementary concept word, protocol supplementary concept word, rare disease supplementary concept word, unique identifier, synonyms]	6825
213	212 not tobacco*.mp. [mp=title, abstract, original title, name of substance word, subject heading word, floating sub-heading word, keyword heading word, organism supplementary concept word, protocol supplementary concept word, rare disease supplementary concept word, unique identifier, synonyms]	6809
214	213 not intercourse.mp. [mp=title, abstract, original title, name of substance word, subject heading word, floating sub-heading word, keyword heading word, organism supplementary concept word, protocol supplementary concept word, rare disease supplementary concept word, unique identifier, synonyms]	6809
215	214 not slum*.mp. [mp=title, abstract, original title, name of substance word, subject heading word, floating sub-heading word, keyword heading word, organism supplementary concept word, protocol supplementary concept word, rare disease supplementary concept word, unique identifier, synonyms]	6807
216	215 not MRI.mp. [mp=title, abstract, original title, name of substance word, subject heading word, floating sub-heading word, keyword heading word, organism supplementary concept word, protocol supplementary concept word, rare disease supplementary concept word, unique identifier, synonyms]	6744
217	216 not multiple sclerosis.mp. [mp=title, abstract, original title, name of substance word, subject heading word, floating sub-heading word, keyword	6742

	heading word, organism supplementary concept word, protocol supplementary concept word, rare disease supplementary concept word, unique identifier, synonyms]	
218	217 not qualitative.mp. [mp=title, abstract, original title, name of substance word, subject heading word, floating sub-heading word, keyword heading word, organism supplementary concept word, protocol supplementary concept word, rare disease supplementary concept word, unique identifier, synonyms]	6163
219	218 not eat*.mp. [mp=title, abstract, original title, name of substance word, subject heading word, floating sub-heading word, keyword heading word, organism supplementary concept word, protocol supplementary concept word, rare disease supplementary concept word, unique identifier, synonyms]	6013
220	219 not blood*.mp. [mp=title, abstract, original title, name of substance word, subject heading word, floating sub-heading word, keyword heading word, organism supplementary concept word, protocol supplementary concept word, rare disease supplementary concept word, unique identifier, synonyms]	5851
221	220 not rehab*.mp. [mp=title, abstract, original title, name of substance word, subject heading word, floating sub-heading word, keyword heading word, organism supplementary concept word, protocol supplementary concept word, rare disease supplementary concept word, unique identifier, synonyms]	5641
222	221 not asthma.mp. [mp=title, abstract, original title, name of substance word, subject heading word, floating sub-heading word, keyword heading word,	5604

	organism supplementary concept word, protocol supplementary concept word, rare disease supplementary concept word, unique identifier, synonyms]	
223	222 not neonat*.mp. [mp=title, abstract, original title, name of substance word, subject heading word, floating sub-heading word, keyword heading word, organism supplementary concept word, protocol supplementary concept word, rare disease supplementary concept word, unique identifier, synonyms]	5602
224	223 not molar.mp. [mp=title, abstract, original title, name of substance word, subject heading word, floating sub-heading word, keyword heading word, organism supplementary concept word, protocol supplementary concept word, rare disease supplementary concept word, unique identifier, synonyms]	5601
225	224 not sedat*.mp. [mp=title, abstract, original title, name of substance word, subject heading word, floating sub-heading word, keyword heading word, organism supplementary concept word, protocol supplementary concept word, rare disease supplementary concept word, unique identifier, synonyms]	5596
226	225 not epidemiolog*.mp. [mp=title, abstract, original title, name of substance word, subject heading word, floating sub-heading word, keyword heading word, organism supplementary concept word, protocol supplementary concept word, rare disease supplementary concept word, unique identifier, synonyms]	5225
227	226 not hygien*.mp. [mp=title, abstract, original title, name of substance word, subject heading word, floating sub-heading word, keyword heading word, organism supplementary concept word, protocol supplementary concept word, rare disease supplementary concept word, unique identifier, synonyms]	5203

228	227 not prison*.mp. [mp=title, abstract, original title, name of substance word, subject heading word, floating sub-heading word, keyword heading word, organism supplementary concept word, protocol supplementary concept word, rare disease supplementary concept word, unique identifier, synonyms]	5188
229	228 not cortisol.mp. [mp=title, abstract, original title, name of substance word, subject heading word, floating sub-heading word, keyword heading word, organism supplementary concept word, protocol supplementary concept word, rare disease supplementary concept word, unique identifier, synonyms]	5179
230	229 not government*.mp. [mp=title, abstract, original title, name of substance word, subject heading word, floating sub-heading word, keyword heading word, organism supplementary concept word, protocol supplementary concept word, rare disease supplementary concept word, unique identifier, synonyms]	5042
231	230 not speech.mp. [mp=title, abstract, original title, name of substance word, subject heading word, floating sub-heading word, keyword heading word, organism supplementary concept word, protocol supplementary concept word, rare disease supplementary concept word, unique identifier, synonyms]	4729
232	231 not FMRI.mp. [mp=title, abstract, original title, name of substance word, subject heading word, floating sub-heading word, keyword heading word, organism supplementary concept word, protocol supplementary concept word, rare disease supplementary concept word, unique identifier, synonyms]	4567
233	232 not EMG.mp. [mp=title, abstract, original title, name of substance word, subject heading word, floating sub-heading word, keyword heading word,	4560

	organism supplementary concept word, protocol supplementary concept word, rare disease supplementary concept word, unique identifier, synonyms]	
234	233 not drug*.mp. [mp=title, abstract, original title, name of substance word, subject heading word, floating sub-heading word, keyword heading word, organism supplementary concept word, protocol supplementary concept word, rare disease supplementary concept word, unique identifier, synonyms]	4426
235	234 not victim*.mp. [mp=title, abstract, original title, name of substance word, subject heading word, floating sub-heading word, keyword heading word, organism supplementary concept word, protocol supplementary concept word, rare disease supplementary concept word, unique identifier, synonyms]	4387
236	235 not stimulant.mp. [mp=title, abstract, original title, name of substance word, subject heading word, floating sub-heading word, keyword heading word, organism supplementary concept word, protocol supplementary concept word, rare disease supplementary concept word, unique identifier, synonyms]	4387
237	236 not marijuana*.mp. [mp=title, abstract, original title, name of substance word, subject heading word, floating sub-heading word, keyword heading word, organism supplementary concept word, protocol supplementary concept word, rare disease supplementary concept word, unique identifier, synonyms]	4385
238	237 not coronary.mp. [mp=title, abstract, original title, name of substance word, subject heading word, floating sub-heading word, keyword heading word, organism supplementary concept word, protocol supplementary concept word, rare disease supplementary concept word, unique identifier, synonyms]	4384

239	238 not heart*.mp. [mp=title, abstract, original title, name of substance word, subject heading word, floating sub-heading word, keyword heading word, organism supplementary concept word, protocol supplementary concept word, rare disease supplementary concept word, unique identifier, synonyms]	4327
240	239 not nutrition*.mp. [mp=title, abstract, original title, name of substance word, subject heading word, floating sub-heading word, keyword heading word, organism supplementary concept word, protocol supplementary concept word, rare disease supplementary concept word, unique identifier, synonyms]	4234
241	240 not borderline personalit*.mp. [mp=title, abstract, original title, name of substance word, subject heading word, floating sub-heading word, keyword heading word, organism supplementary concept word, protocol supplementary concept word, rare disease supplementary concept word, unique identifier, synonyms]	4228
242	241 not brain injur*.mp. [mp=title, abstract, original title, name of substance word, subject heading word, floating sub-heading word, keyword heading word, organism supplementary concept word, protocol supplementary concept word, rare disease supplementary concept word, unique identifier, synonyms]	4223
243	242 not cystic fibrosis.mp. [mp=title, abstract, original title, name of substance word, subject heading word, floating sub-heading word, keyword heading word, organism supplementary concept word, protocol supplementary concept word, rare disease supplementary concept word, unique identifier, synonyms]	4221

244	243 not feed*.mp. [mp=title, abstract, original title, name of substance word, subject heading word, floating sub-heading word, keyword heading word, organism supplementary concept word, protocol supplementary concept word, rare disease supplementary concept word, unique identifier, synonyms]	3995
245	244 not arthritis.mp. [mp=title, abstract, original title, name of substance word, subject heading word, floating sub-heading word, keyword heading word, organism supplementary concept word, protocol supplementary concept word, rare disease supplementary concept word, unique identifier, synonyms]	3993
246	245 not aborigin*.mp. [mp=title, abstract, original title, name of substance word, subject heading word, floating sub-heading word, keyword heading word, organism supplementary concept word, protocol supplementary concept word, rare disease supplementary concept word, unique identifier, synonyms]	3984
247	246 not surviv*.mp. [mp=title, abstract, original title, name of substance word, subject heading word, floating sub-heading word, keyword heading word, organism supplementary concept word, protocol supplementary concept word, rare disease supplementary concept word, unique identifier, synonyms]	

Appendix 2: Included outcome measures

Impairment Rating Scale (IRS): Measures impairment across domains of functioning and overall need for treatment. It is a 7-point visual analogue scale to show the child's functioning and impairment on a scale ranging from 0 (not a problem at all. Definitely does not need treatment of special services) to 6 (extreme problem. Definitely needs treatment and special services). The

IRS also includes measures of the impact of child's peer relationships, relationships with their parents, family functioning and overall impairment (Fabiano et al., 2006).

Strengths and Difficulties Questionnaire (SDQ): A five-step response scale from 1 (does not apply at all) to 5 (applies very well) and is a brief behavioral screening questionnaire for 3-16-year olds. There are five domains: emotional symptoms, conduct problems, hyperactivity/inattention, peer relationship problems, prosocial behavior. Ostberg et al (2012) used a version of the SDQ that is validated in Sweden (Malmberg et al., 2003; Smedje et al., 1999). The SDQ can be parent, child or teacher rated (Malmberg et al., 2003; Smedje et al., 1999).

Social Skills Improvement System (SSIS); social skills subscale – The parent and teacher reported SSIS social skills sub scale includes 46 items around communication, cooperation, assertion, responsibility, empathy and self-control skills. Each item is rated on a 4-point scale; never, seldom, often or almost always (F. Gresham & Elliott, 2007).

Social Skills Rating System (SSRS): A 55 item (parent reported) or 57 item (teacher reported) scale whereby each point is rated on a three-point metric (never, sometimes, very often) and aims to evaluate social behaviors of children and adolescents. There are five subscales of the SSRS: cooperation, assertion, responsibility, empathy and self-control (F. M. Gresham & Elliott, 1990).

Quality of Play Questionnaire: Parents answer 18 questions on a 4 points scale from 0-3 (not at all, just a little, pretty much and very much) regarding how their child and friend interact during

the most recent playdate and involves two subscales: conflict and disengagement *(Frankel & Mintz, 2008)*.

University of California Irvine Social Skills Scale (UCI): The UCI is a 10-item rating scale constructed specifically for Pfiffner et al's (1997) study and aims to measure social behaviors taught in the treatment groups. Parents rate to the extent their child follows game rules, maintains participation in an activity or game, says nice things to others, follows directions, uses assertive communication, ignores teasing, uses problem solving, recognises others' feelings, and deals with anger appropriately. Each skill is rated on a 5-point scale (1:never – 5: most or all of the time) (Pfiffner & McBurnett, 1997).

Test of Social Skills Knowledge: Designed to assess children's knowledge about social skills taught during the class in Pfiffner et al's (1997) study. This measure is administered individually to each child and contains six questions including "What are some good examples of good sportsmanship?" "How would you show you are being assertive?" "What are some ways to deal with anger?" Children are asked to respond verbally, and an interviewer records the responses. Responses are later allocated a score (1: no or inaccurate response, 2: partial response, 3 full accurate response).

Test of Life Skills Knowledge: The Test of Life Skills Knowledge is designed to assess social and organizational skills taught in the child group (Pfiffner & Mikami, 2005). It is a 10-item child reported test including questions such as "What should you do if someone is teasing you?" "How would you show that you are being assertive?" "What are some ways to make a new friend?"

Child responses are recorded and responses are later rated on a 3-point scale (1: no or inaccurate response, 2: partial response, 3: full accurate response) (Pfiffner & McBurnett, 1997).

Social Competence Scale (SCS): Consists of 12 items to assess parent perceptions of child's positive social behaviors (e.g. resolving peer problems, understanding others, sharing, being helpful, listening and emotional regulation) (McMahon et al., 1999).

Wally Problem Solving Scale: The Wally Problem Solving Test (Webster-Stratton, 1990) aims to measure children's problem-solving skills by assessing their responses to hypothetical conflict situations. The summary score indicates the ratio of positive to negative strategies the child states.

Test of Playfulness: Aims to examine children's play skills, through observation, in peer-to-peer play interactions pre intervention and one-month post intervention. It contains nine items that reflect social skills: initiating interactions, negotiating, sharing, supporting one another, time engaged in social interactions, intensity of involvement with another in a social interaction, social skills when interacting with another, giving verbal and non-verbal cues and responding to other verbal and non-verbal cues (Bundy et al., 2016).

Appendix 3: Details of the CRoB quality assessment.

**Author, year**	**Random sequence generation**	**Allocation concealment**	**Blinding of participants and personnel**	**Blinding of outcome assessment**	**Incomplete outcome data**	**Selective reporting**	**Overall risk**
Chacko, 2009 (Chacko et al., 2009)	Unclear - random sequence generation not reported	Unclear - allocation concealment not reported	High risk - participants and personnel unable to be blinded due to nature of intervention	Unclear - outcome assessment blinding not reported	Low risk - 5/120 (5%) lost to follow up overall. Break down of drop-out rates per study arm not reported.	Low risk - no protocol reported but primary and secondary measures assessed, and results presented	Unclear
Ferrin 2016 (Ferrin et al., 2016)	Low risk - computerised programme used by Clinical Trials Unit	Unclear - allocation concealment not reported	High risk - participants and personnel unable to be blinded due	Low risk - researchers collecting and entering data were	Low risk: Intervention: 3/35 ((1.05%) and control: 4/34	Low risk - no protocol reported but primary and	Unclear

			to nature of intervention	blind to group allocation	(1.36%) lost to follow up	secondary measures assessed and results presented	
CLAS Study (Haack et al., 2017; Pfiffner et al., 2014)	Unclear - random sequence generation not reported	Unclear - allocation concealment not reported	High risk - participants and personnel unable to be blinded due to nature of intervention	Unclear - outcome assessment blinding not reported	Low risk: Intervention: 5/74 (3.7%); PFT: 1/74 (0.74%) and Control: 6/51 (3.06%) lost to follow up. Missing values reported pre treatmnet (0.8%), posttreatment (3.3%) and at follow up (10.6%).	Low risk - no protocol reported but primary and secondary measures assessed and results presented	Unclear

Mikami, 2010 (Mikami et al., 2010)	Unclear - sufficient detail of random sequence generation not reported	Unclear - allocation concealment not reported	High risk - participants and personnel unable to be blinded due to nature of intervention	Unclear - outcome assessment blinding not reported	Low risk: PFC: 4/32 (1.28%); ADHD Control: 1/30 (0.3%); Normative comparison 0/62 (0%) lost to follow up	Low risk - no protocol reported but primary and secondary measures assessed and results presented	Unclear
Ostberg, 2012 (Östberg & Rydell, 2012)	Unclear - random sequence generation not reported	Unclear - allocation concealment not reported	High risk - participants and personnel unable to be blinded due to nature of intervention	Unclear - outcome assessment blinding not reported	Low risk - Intervention: 7/36 (2.5%); Control: 2/34 (0.68%) lost to follow up.	Low risk - no protocol reported but primary and secondary measures assessed and results presented	Unclear

Pfiffner, 1997 (Pfiffner & McBurnett, 1997)	Unclear - random sequence generation not reported	Unclear - allocation concealment not reported	High risk - participants and personnel unable to be blinded due to nature of intervention	Unclear - outcome assessment blinding not reported	Low risk -no participants were lost to follow up	Low risk - no protocol reported but primary and secondary measures assessed and results presented	Unclear
Pfiffner, 2007 (Pfiffner et al., 2007)	Unclear - sufficient detail of random sequence generation not reported	Unclear - allocation concealment not reported	High risk - participants and personnel unable to be blinded due to nature of intervention	Unclear - outcome assessment blinding not reported	Low risk - Intervention 7/36 (2.5%); Control: 8/33 (2.64%) lost to follow up	Low risk - no protocol reported but primary and secondary measures assessed and results presented	Unclear

CLS Study (L. J. Pfiffner et al., 2016; Pfiffner et al., 2018)	Unclear - sufficient detail of random sequence generation not reported	Low risk - randomised by a statistician	High risk - participants and personnel unable to be blinded due to nature of intervention	High risk - assessors not blinded	Low risk - Intervention 0/72 (0%) and control: 1/63 (0.63%) lost to follow up.	Low risk - no protocol reported but primary and secondary measures assessed and results presented	High risk
IY Study (Webster-Stratton et al., 2013; Webster-Stratton et al., 2011)	Unclear - random sequence generation not reported	Unclear - allocation concealment not reported	High risk - participants and personnel unable to be blinded due to nature of intervention	Unclear - outcome assessment blinding not reported	Low risk - Intervention: 2/49 (0.98%); control: 3/50 (1.5%) lost to follow up	Low risk - no protocol reported but primary and secondary measures assessed and results presented. Reference	Unclear

						made to 2011 paper in 2013 paper with regards to study procedures.	
Wilkes-Gillan, 2016 (Wilkes-Gillan et al., 2016)	Low risk - opaque envelopes used	Low risk - academic staff member outside of study team allocated groups	High risk - participants and personnel blinded at baseline but then they were unable to be blinded due to nature of intervention	Low risk - assessors blinded to treatment allocation	Low risk: Intervention 1/16 (0.16%); control: 1/15 (0.15%) lost to follow up.	Low risk - trial protocol reported, and all primary and secondary measures assessed and results presented.	Low risk

Of the included RCTs, eight did not report the random sequence generation (Chacko et al., 2009; Haack et al., 2017; Mikami et al., 2010; Östberg & Rydell, 2012; Pfiffner et al., 2014; Pfiffner & McBurnett, 1997; Pfiffner et al., 2007; Pfiffner et al., 2016; Pfiffner et al., 2018; Webster-

Stratton et al., 2013; Webster-Stratton et al., 2011) and were judged to have an unclear risk of bias for this domain. Two studies were judged as having a low risk for this domain as they used opaque envelopes for randomisation (Wilkes-Gillan et al., 2016) or a computerised randomisation programme (Ferrin et al., 2016).

Eight of the eleven included RCTs were judged as having an unclear risk of boas for allocation concealment as this information as not reported (Chacko et al., 2009; Ferrin et al., 2016; Haack et al., 2017; Mikami et al., 2010; Östberg & Rydell, 2012; Pfiffner et al., 2014; Pfiffner & McBurnett, 1997; Pfiffner et al., 2007; Webster-Stratton et al., 2013; Webster-Stratton et al., 2011). The CLS and IY studies were judged has having a low risk of bias for this domain as randomisation was reported to be conducted by a statistician (Pfiffner et al., 2016; Pfiffner et al., 2018) or an individual outside the study team (Wilkes-Gillan et al., 2016).

All included RCTs were judged as having a high risk of bias for the blinding of participants as due to the nature of these studies, blinding was not possible (Chacko et al., 2009; Ferrin et al., 2016; Haack et al., 2017; Mikami et al., 2010; Östberg & Rydell, 2012; Pfiffner et al., 2014; Pfiffner & McBurnett, 1997; Pfiffner et al., 2007; Pfiffner et al., 2016; Pfiffner et al., 2018; Webster-Stratton et al., 2013; Webster-Stratton et al., 2011; Wilkes-Gillan et al., 2016).

Seven included studies did not report outcome assessment blinding and were therefore judged as having an unclear risk of boas for this domain (Chacko et al., 2009; Haack et al., 2017; Mikami et al., 2010; Östberg & Rydell, 2012; Pfiffner et al., 2014; Pfiffner & McBurnett, 1997; Pfiffner et al., 2007; Webster-Stratton et al., 2013; Webster-Stratton et al., 2011). One study was judged has having a high risk of bias for this domain as the assessor was not blinded (Pfiffner et al.,

2016; Pfiffner et al., 2018) and the remaining two studies a low risk of bias as outcome assessment was reported to be blinded (Ferrin et al., 2016; Wilkes-Gillan et al., 2016).

All included studies were reported to have a low risk of bias for the incomplete outcome data domain as they all reported less than a 20% drop out rate (Chacko et al., 2009; Ferrin et al., 2016; Haack et al., 2017; Mikami et al., 2010; Östberg & Rydell, 2012; Pfiffner et al., 2014; Pfiffner & McBurnett, 1997; Pfiffner et al., 2007; Pfiffner et al., 2016; Pfiffner et al., 2018; Webster-Stratton et al., 2013; Webster-Stratton et al., 2011; Wilkes-Gillan et al., 2016).

All included studies were reported to have a low risk of bias for the selective reporting domain as they all reported all primary and secondary outcome measure results (Chacko et al., 2009; Ferrin et al., 2016; Haack et al., 2017; Mikami et al., 2010; Östberg & Rydell, 2012; Pfiffner et al., 2014; Pfiffner & McBurnett, 1997; Pfiffner et al., 2007; Pfiffner et al., 2016; Pfiffner et al., 2018; Webster-Stratton et al., 2013; Webster-Stratton et al., 2011; Wilkes-Gillan et al., 2016).

Therefore, eight of the ten included studies were judged as having an overall unclear risk of bias (Chacko et al., 2009; Ferrin et al., 2016; Haack et al., 2017; Mikami et al., 2010; Östberg & Rydell, 2012; Pfiffner et al., 2014; Pfiffner & McBurnett, 1997; Pfiffner et al., 2007; Webster-Stratton et al., 2013; Webster-Stratton et al., 2011), one was judged to have a high risk of bias (Pfiffner et al., 2016) and the remaining study an overall low risk of bias (Wilkes-Gillan et al., 2016).

Appendix 4: Detail of Interventions

The descriptions of the interventions below are as described by the corresponding authors.

Chacko, 2009

Strategies to Enhance Positive Parenting (STEPP) program. This was a manualised 9-week programme held in large groups for 2.5 hours each week. It included videotaped vignettes, therapist-facilitated questions, group discussions, modelling, and role-plays by parents. Children participated in a group social skills program.

Open-ended questions were asked of single mothers regarding their expectations about theirs and their child's involvement in treatment (e.g., What role do you think you will have in treatment? In what way do you think your child will be involved in treatment?). Single mothers were also asked open-ended questions regarding their expectations about the rate and potency of treatment-related improvements for their child (e.g., How fast do you expect to observe improvements in your child's behavior?) and about their attributions regarding locus of control of their child's behavior (e.g., What do you think causes your child to misbehave?) and the effect of their parenting (e.g., In what ways have you seen you parenting make a difference?). Practical barriers (e.g., childcare, transportation) to ongoing involvement were addressed and solutions to these barriers were developed.

The STEPP program also incorporated a subgroup, coping-modeling, problem- solving format within the traditional large-group format to improve social support between parents and to increase participation among parents. The STEPP program also incorporated a systematic, problem- solving treatment method to address parent-initiated problems (e.g., time management, conflicts with relatives) that may either interfere with their parenting or affect parents' psychosocial functioning. In addition, the STEPP program incorporates parent–child interactions within the children's social skills group to enhance parenting skill acquisition and a

child motivation enhancement within the children's social skills group to provide children incentives for attaining within- session and home-based behavioral goals.

Ferrin, 2016

The psychoeducation group included five groups of seven to ten families, who received six sessions of 2 hour at weekly intervals. Families were primarily educated on the disorder; they were briefly introduced to a range of behavioral strategies for managing ADHD symptoms and reducing defiant behavior during the last three sessions. The integrity of the psychoeducation sessions was guaranteed by a manual that clearly outlined all the procedures to be used in the intervention. Two experienced child and adolescent psychiatrists and one psychologist conducted all the sessions. Sessions were audiotaped and an independent person using a checklist ensured that the different groups received an equivalent set of information. At the end of each session, a handout was delivered and parents were assigned some short additional homework to prepare for the next session.

Pfiffner, 2007; CLAS Study, (Pfiffner, 2014; Haak, 2017)

Child Life and Attention Skills (CLAS) treatment involved parent and child skills training that included:

Teacher consultation: This involved an overview of behavioural interventions and ADHD classroom-based accommodations. Target behaviours were identified and the skills that were taught in the child group were also shared with teachers so they could reinforce the behaviours in the classroom.

Parent training: This involved providing the parents with an overview of ADHD, ADHD management strategies and a "home challenge" that involved specific target behaivors.

Child skills training: This was divided into modules that focussed on skills for independence (e.g., academic study, organisaiton, self-care, daily living skills) and skills for social competence.

Mikami, 2010

Parental Friendship Coaching (PFC) consisted of eight 90-minute group sessions, delivered once-weekly, involving five to six parents and led by two clinicians. Sessions were manualized, although minor changes to content occurred based on parent feedback. Each PFC session began with a review of homework from the previous week. Then, the target parental coaching strategies of the week (e.g. building a positive parent-child relationship, using active listening, debriefing with child after a playdate, review and future directions) were explained using handouts, activities, and role plays. Parents were encouraged to bring up ways in which strategies could be tailored to their child's specific needs. Group viewing of videotapes of each parent's interaction with his/her child during the playgroups was used as a teaching tool.

Ostberg, 2012

A slightly modified version of Barkley's parent training programme adapted to Swedish circumstances and conditions. A parallel and similar programme for teachers was constructed with the goal to address the child's two major contexts, home and school at the same time. The intervention is manual based, as is training for group leaders. Parents met for 10 weekly 2-hour sessions, and teachers met for eight sessions, with parents and teachers of about eight children per group. The sessions focussed upon information about neuropsychiatric problems and on participants learning to use reinforcements, to solve problems and to communicate with the

child. Home assignments and discussions of these were part of the programme, and a structure for the co-operation between home and school was formed.

Pfiffner, 1997

Children received eight 90-minute sessions during consecutive weeks. Sessions were taught by the same two therapists. During each session, leaders taught skill modules using brief didactic instruction, symbolic (puppets, stuffed animals) and in vivo modelling, role playing and behavioral rehearsal. Six modules were covered: good sportsmanship, accepting consequences, assertiveness, ignoring provocation, problem solving, recognising and deadline with feelings. Sessions were structured in the following way: Review of social skill used in the previous week, discussion of "good sports bucks" earned at home for targeted social skills. Second, leaders introduced the "skill of the week" and then reviewed how, when and why to use the skill with child participation in a group challenge game. Handouts were distributed to parents at the end of each week.

CLS Study (Pfiffner, 2016; 2018)

The Collaborative Life Skills Training (CLS) involved three components:

Classroom Component: Teachers attended one 1-hour orientation session, one 30-minute troubleshooting meeting and two to three individual 30-minute meetings attended by the parent, the student, and the student's individual teacher. The classroom intervention consisted of a school– home daily report card (classroom challenge), homework plan, and classroom accommodations as needed (e.g., preferential seating, targeted use of praise, providing prompts to improve student compliance). Each student's CC included two to three target behaviors (e.g., academic work, classroom deportment, social interactions) rated up to three

times per day. Points earned for meeting target goals were exchanged for daily home rewards and brought to the child group each week for group-based reinforcement. Target behaviors were refined throughout the intervention period during the individual meetings.

Parent Component: Parents attended ten 1-hour group sessions. Modules taught skills covered by traditional parent training programs, including effective use of commands, rewards, and discipline, plus strategies covered in the child group (e.g., homework time, organization, independence in completing daily routines, peer interactions, and social skills) and stress management for parents. Families developed a homework plan and home challenges targeting child skills. They also learned skills for supporting the CC at home.

Child Skills Component: Children attended nine 40-minute group sessions during the school day and two celebratory parties with parents, teachers, and students. Modules targeted social functioning and independence. Social skills modules included good sportsmanship, accepting consequences, assertion, dealing with teasing, problem solving, self-control, and friendship making. Independence modules included homework skills, completing chores and tasks independently, and establishing and following routines. Activities accommodated developmental needs (e.g., having older children take more of a leader/helper role in groups, providing age-appropriate examples of skill use). Skills were taught through didactic instruction, behavior rehearsal, and in vivo prac- tice. A reward-based contingency management program was used to manage child behavior, encourage active group participation, and reinforce new

skills. To facilitate generalization, children earned tokens and rewards for accomplishing target goals at home and school.

IY Study (Webster Stratton 2011; 2013)

The Incredible Years (IY) programme included both a parent and a child program. The IY parent training intervention consisted of 20 weekly, two-hour sessions conducted with six families per group. The newest version of the basic IY preschool curriculum (revised 2008) was offered. This updated version of the program had new curriculum material focusing on academic, persistence, social and emotional coaching, establishing predictable household routines and schedules, emotion regulation strategies, and teaching children to problem solve. This version of the program included new vignettes showing children with ADHD in order to enhance parental understanding of how to respond effectively to these children and understand their developmental levels and temperament. Additional sessions from the IY advance parent curriculum included problem solving between adults and with teachers regarding child behavior plans, and strategies to build family interpersonal support, reduce depression, and manage anger.

The IY Dinosaur training program (for the children) was held at the same time as the parent program. Program topics included following group rules, identifying and articulating feelings, problem solving, anger management, friendship skills, and teamwork. Each two-hour session consisted of three short circle times and three to four planned activities to reinforce concepts presented in circle time. Therapists used coaching methods during unstructured play times to encourage appropriate peer interactions and targeted social and emotional skills.

Wilkes Gillen, 2016

Children watched videos of themselves playing and received feedback from therapists. Therapists helped children remember 3 key things for when they next enter the playroom. During play, the therapist modelled desired pro-social skills; sharing, perspective taking, problem solving, negotiating and responding to playmates verbal and non verba cues. Childre later played without therapist support.
